# Revision of the Eocene ‘*Platyrhina*’ species from the Bolca Lagerstätte (Italy) reveals the first panray (Batomorphii: Zanobatidae) in the fossil record

**DOI:** 10.1080/14772019.2020.1783380

**Published:** 2020-07-13

**Authors:** Giuseppe Marramà, Giorgio Carnevale, Kerin M. Claeson, Gavin J. P. Naylor, Jürgen Kriwet

**Affiliations:** a Università degli Studi di Torino, Dipartimento di Scienze della Terra, via Valperga Caluso 35, 10125 Torino, Italy;; b University of Vienna, Department of Palaeontology, Althanstrasse 14, 1090 Vienna, Austria;; c Philadelphia College of Osteopathic Medicine, Philadelphia, PA 19103, USA;; d University of Florida, Florida Museum of Natural History, 1659 Museum Road, 32611 Gainesville, USA

**Keywords:** †*Eoplatyrhina* gen. nov., †*Plesiozanobatus* gen. nov., phylogenetic analysis, Batoidea, Eocene, Bolca

## Abstract

The fossil-Lagerstätte of Bolca (Italy) is well known for the diversity and exquisite preservation of its bony and cartilaginous fishes documenting tropical shallow-water marine environments associated with coral reefs in the western Tethys during the early Eocene. In this study, the taxonomic, systematic and phylogenetic position of two batoid species traditionally assigned to the living thornback ray genus *Platyrhina* is re-evaluated. †*Platyrhina bolcensis* Heckel, 1851 is recognized as a separate species of the Platyrhinidae because of its plate-like antorbital cartilage with an irregular outline and a small horn on the nasal capsules. Also, the rostral cartilage does not reach the anterior border of the disc. Support for the placement of this species within the new genus †*Eoplatyrhina* gen. nov. is based on a combination of morphological and meristic features (e.g. nasal capsules at right angles to the rostrum; large space between the hyomandibulae and mandibular arch; approximately 132 vertebral centra; 15–16 rib pairs; 81–87 pectoral radials; 18–21 pelvic radials; short, straight and stout claspers; 40–50 caudal-fin radials; thorns absent). A second species, †*Platyrhina egertoni* (De Zigno, 1876), is more closely related to the living panray *Zanobatus* than *Platyrhina* and is assigned here to †*Plesiozanobatus* gen. nov. because of a combination of characters that support its placement within the family Zanobatidae (tail stout and short, distinctly demarcated from disc; two dorsal fins and complete caudal fin; small dermal denticles and scattered thorns covering disc and tail; rostral cartilage absent; nasal capsules without horn-like processes; mesopterygium absent). The systematic position of a third taxon, †*Platyrhina gigantea* (Blainville, 1818), is currently impossible to establish due to the poor preservation of the only known specimen, and therefore we propose to consider it a *nomen dubium*. Palaeoecological and biogeographic features of the Eocene platyrhinids and zanobatids from Bolca are also discussed.

http://zoobank.org/urn:lsid:zoobank.org:pub:B4C7A979-7972-409B-B489-A6DDD5E35FE5

## Introduction

The Ypresian Konservat-Lagerstätte of Bolca, in north-eastern Italy, is one of the few Palaeogene deposits where fossils of cartilaginous fishes (Chondrichthyes) are exquisitely preserved (Marramà *et al*. 2018c). Individuals include complete and fully articulated skeletal remains, which is the exception in the fossil record with chondrichthyans mostly being represented by isolated teeth (Cappetta 2012). Recent studies have contributed to the knowledge of the taxonomy and systematic position of the cartilaginous fishes from the Pesciara and Monte Postale sites of Bolca, which include about a dozen species-level taxa belonging to a variety of holocephalian, selachian and batoid lineages (Fanti *et al.* 2016, 2019; Marramà *et al*. 2018a, b, c, 2019a, b, c, d;). These batoids are represented by electric rays (Torpediniformes), guitarfishes (Rhinopristiformes), stingrays (Myliobatiformes) and three batoid species that were historically assigned to the thornback ray genus *Platyrhina* Müller & Henle, 1838: †*P. bolcensis* Heckel, 1851, †*P. egertoni* (De Zigno, 1876) and †*P. gigantea* (Blainville, 1818). The last account of these three batoid species was provided at the end of the nineteenth century by Jaekel (1894) in his comprehensive review of the elasmobranch fishes from Bolca known at that time. In this paper, we redescribe and re-evaluate the systematic position of the fossil material from Bolca traditionally assigned to *Platyrhina* in the context of our current understanding of platyrhinid phylogenetics.

The higher taxonomic placement and interrelationships of the families Platyrhinidae and Zanobatidae within batoid fishes are still debated today. According to morphological studies, these families are traditionally considered successive sister taxa to the stingray order Myliobatiformes (McEachran *et al*. 1996; McEachran & Aschliman 2004; Aschliman *et al*. 2012a; Villalobos-Segura *et al*. 2019). Conversely, molecular analyses place the Platyrhinidae as sister taxon to the electric ray order Torpediniformes (Aschliman *et al*. 2012b; Naylor *et al*. 2012; Bertozzi *et al*. 2016; Last *et al*. 2016), whereas the Zanobatidae are regarded either as sister to Myliobatiformes (Aschliman *et al*. 2012b; Bertozzi *et al*. 2016; Last *et al*. 2016) or as a member of the order Rhinopristiformes (Naylor *et al*. 2012).

The fossil record of platyrhinids is very poor compared to the other batoid lineages, possibly because their isolated teeth are often misidentified and assigned to the genus *Rhinobatos* (Claeson *et al*. 2013). Fossil platyrhinids can be traced back to the Late Cretaceous and include extinct genera represented by articulated skeletal remains, like †*Tethybatis* from the Campanian/Maastrichtian of southern Italy (Carvalho 2004) and †*Tingitanius* from the Turonian of Morocco (Claeson *et al*. 2013), isolated teeth of †*Cretaplatyrhinoidis* and †*Pseudoplatyrhina* from the Turonian–Santonian of the Anglo-Paris Basin (Guinot *et al*. 2012), and a few occurrences of *Platyrhina* and *Platyrhinoidis* teeth from the Eocene of Egypt (Underwood *et al*. 2011) and Pleistocene of California (Long 1993). †*Platyrhina ypresiensis and* †*P. dockeryi* from the Eocene of Belgium and the USA have been recently transferred to the myliobatiform genus †*Hypolophodon* Cappetta, 1980 (Cappetta 2012; Case *et al*. 2015). †*Britobatos primarmatus* (Woodward, 1889), from the Santonian of Lebanon, was suggested to belong to the Platyrhinidae by Brito & Dutheil (2004), although Claeson *et al*. (2013) excluded this taxon from this family, instead placing it as a sister to the family. †*Protoplatyrhina*, based on isolated teeth from the Late Cretaceous of North America, was considered a possible ancestor of *Platyrhina* by Case (1978). However, Cappetta (1987, 2012) rejected this hypothesis and considered †*Protoplatyrhina* to be a rhinobatoid of family *incertae sedis*. To our knowledge, the family Zanobatidae has never been recognized in the fossil record until now.

## Geological setting

**Figure 1.**
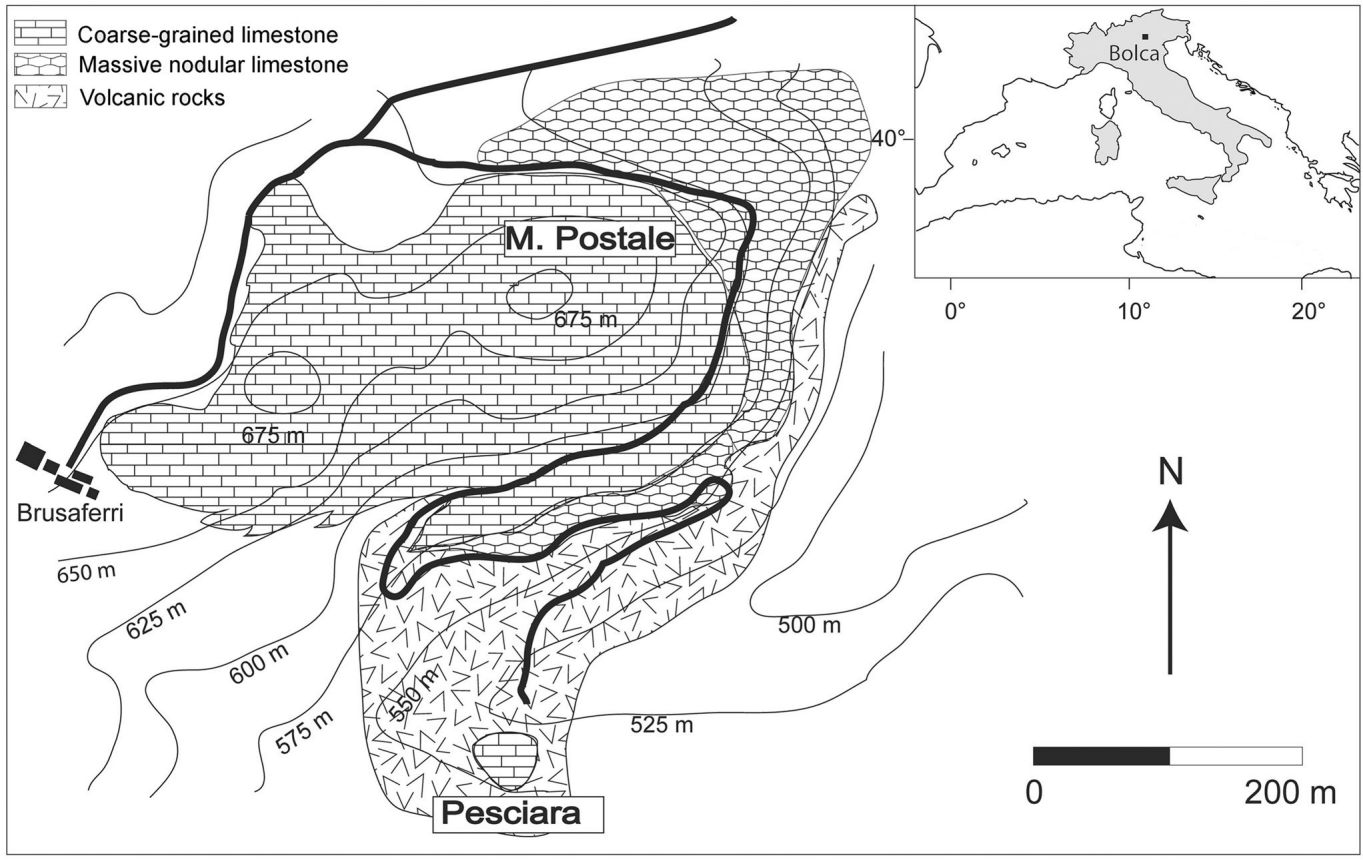
Schematic geological map showing the location of the two main Lagerstätten of Bolca. Adapted from Trevisani (2015) and Marramà *et al*. (2016).

Lithological features, museum catalogue registers and information from the literature suggest that all †‘*Platyrhina*’ *bolcensis* specimens come from the Monte Postale site, whereas specimens of †‘*P*.’ *egertoni* and †‘*P*.’ *gigantea* are from the Pesciara site; these are two of the main fossiliferous deposits of the Bolca Konservat-Lagerstätte located in Verona Province, north-eastern Italy (Fig. 1). The Pesciara and Monte Postale sediments represent shallow-water Eocene sequences deposited on the Lessini Shelf, a palaeogeographical feature of the Southern Alps that was uplifted during the Alpine orogeny, acting as an area of deposition of shallow-water carbonates (Doglioni & Bosellini 1987; Bosellini 1989).

The Pesciara site consists of a limestone outcrop, about 20 m thick, surrounded by volcanic deposits and comprising a rhythmic alternation of finely laminated micritic limestones with fishes, plants and grainstone-bearing benthic fossils (Papazzoni & Trevisani 2006). Based on their larger benthic foraminiferan content, the Pesciara fossiliferous sediments were assigned to the †*Alveolina dainelli* Zone, corresponding to the late Cuisian (late Ypresian, between 48.96 and 48.5 Ma; Papazzoni & Trevisani 2006; Papazzoni *et al*. 2014). Quantitative palaeoecological analyses suggest that the Pesciara fish assemblage was characterized by a sharp oligarchic structure dominated by zooplanktivorous fishes, whereas the taphonomic features support the hypothesis that the fossiliferous sediments accumulated in a shallow intraplatform basin in which anoxic conditions and the development of a microbial mat at the bottom promoted the high-quality preservation of the fossils (Papazzoni & Trevisani 2006; Marramà *et al*. 2016).

The uppermost part of the Monte Postale succession consists of more than 130 m of massive grainstones that alternate with massive coralgal limestones and laminated wackestones with fishes and plants similar to those of the Pesciara site, although the fossiliferous laminites of the latter appear to be slightly younger (e.g. Vescogni *et al*. 2016; Papazzoni *et al*. 2017). Evidence of a coralgal rim, lagoonal deposits, and fore-reef systems were detected for the Monte Postale palaeobiotope (Vescogni *et al*. 2016). This interpretation is also supported by quantitative palaeoecological and taphonomic studies of the Monte Postale fish assemblage, which revealed a high degree of disarticulation of fish skeletons, unimodal dispersion of the elements, and bioturbations, which are interpreted as the result of periodic oxic bottom conditions (Marramà *et al*. 2016). The fossiliferous strata of the Monte Postale span the entire NP 13 (= CNE 5) calcareous nannoplankton zones (Papazzoni *et al*. 2017), corresponding to a large part of the Shallow Benthic Zone (SBZ) 11 in the time interval between 50.5 and 48.96 Ma.

## Material and methods

The present study is based on three nearly complete and articulated specimens traditionally referred to †‘*Platyrhina*’ *bolcensis*, six specimens of †‘*P.*’ *egertoni* and a single individual of †‘*P.*’ *gigantea*. The specimens are currently housed in the Museo Civico di Storia Naturale di Verona, Museo di Geologia e Paleontologia dell’Università degli Studi di Padova, Museo Geologico Giovanni Capellini, Università degli Studi di Bologna, Muséum National d’Histoire Naturelle, Paris, and Museum für Naturkunde, Berlin. Some of the specimens were examined under ultraviolet light in order to distinguish the preserved skeletal and soft tissues from grout or pigments. Measurements were taken to the nearest 0.1 mm. Osteological terminology primarily follows Carvalho (2004), Aschliman *et al*. (2012a) and Claeson *et al*. (2013). A dagger (†) preceding a taxon name is used to indicate that it is extinct.

Specimens from Bolca were treated as operational terminal taxa and added to the morphological data set of Villalobos-Segura *et al*. (2019), which in turn was compiled from the matrices of Aschliman *et al*. (2012a) and Claeson *et al*. (2013) (Supplemental material, File 1; Appendix 1). The original characters 5, 7, 52, 72 and 91 of Villalobos-Segura *et al*. (2019) were excluded because they were found to be uninformative. All the codings were checked and some were corrected based on new observations or according to the most up-to-date literature. The dataset was further concatenated with the molecular matrix of Aschliman *et al.* (2012b) to produce a mixed-data matrix, the subject of a second phylogenetic analysis following on from a morphology-only analysis.

The Bolca morphological matrix also differs from that of Villalobos-Segura *et al.* (2019) by taking into account four potential outgroups relative to the ingroup clade, crown Batoidea: *Chimaera*, *Heterodontus*, Hexanchidae and *Squalus*. The morphological matrix based on Villalobos-Segura *et al*. (2019) included Hexanchidae and Chimaeridae as outgroups, but while building the mixed-data matrix, we did not have molecular data aligned for Hexanchidae; however, we did have data for *Heterodontus* and *Squalus*. We could also generate morphological codings based on Aschliman *et al*. (2012a) and personal observations for *Heterodontus* and *Squalus*, so we included these in both the morphological and mixed-data matrices. †*Britobatos primarmatus* is excluded from our analyses because some characters were re-coded without explanation by Villalobos-Segura *et al*. (2019) and were discordant with respect to the codings of Brito & Dutheil (2004) and Claeson *et al*. (2013), suggesting that a revision of the fossil material is needed. We added 14 additional characters mostly taken from Aschliman *et al*. (2012a) and Claeson *et al*. (2013). Additional characters and updated coding are explained in the Supplemental material, File 1. The morphological matrix was compiled in Mesquite v. 3.03 (Maddison & Maddison 2008), and the phylogenetic analysis was performed in PAUP v. 4.0a (build 166) utilizing a heuristic search with stepwise addition, amb(-) and 1000 random addition replicates (Swofford 2002). All characters are unordered and given equal weight. Tree length, consistency and retention indices, and Bremer support were subsequently calculated for the strict consensus tree.

Additional variations of the morphological matrix concern character 5 (calcified suprascapulae: [0] absent, [1] present and independent). Compagno (1999) considered the scapular process to be the unsegmented dorsomedial projection from the scapulocorocoid, and articulating with the scapular process is another small cartilage, the suprascapula. In a paper by Da Silva *et al.* (2018, figs 1A, 3B) the scapula is defined as the projection from the scapulocorocoid in sharks (e.g. *Squalus* and *Heterodontus*) with a segmented scapular process, while in batoids, the scapular process is a non-segmented projection. To account for this variation, we have done the following: (1) retained the coding for character 5, as a suprascapula is present according to Compagno (1999), adjusting all correlated characters (designated CH coding; Supplemental material, File 2); (2) changed the coding of character 5 to follow Da Silva *et al*. (2018) and adjusted all correlated characters (suprascapular absent in *Squalus* and *Heterodontus*, designated DS coding; Supplemental material, File 3); and (3) ran a parsimony analysis excluding *Heterodontus* and *Squalus* as in Villalobos-Seguera *et al.* (2019), updating codings for all characters. In addition, given controversy over the developmental states of the hypobranchial 2 cartilage described by Miyake & McEachran (1991), we also ran an analysis excluding character 85 (hypobranchial shape: [0] straight and segmented, [1] loop/horseshoe shaped, [2] bilateral fused plates, [3] medially fused plates).

The revised morphological data sets (CH and DS codings) were concatenated with the molecular matrix published by Aschliman *et al*. (2012b) to produce the mixed-data matrix for total evidence analyses. When combining the morphological and molecular data sets, we opted to reduce the amount of missing data by excluding one out of three electric ray taxa, three out of eight skates and nine out of 16 stingray taxa originally included in Aschliman *et al*. (2012b). There is high support for the monophyly of the clades Torpediniformes, Rajiformes and Myliobatiformes (e.g. Aschliman *et al*. 2012a, b; Claeson 2014). From our original morphological matrix, taxa with insufficient molecular sequences were excluded: the outgroup taxon Hexanchidae, and the electric ray taxa *Hypnos*, *Narke* and *Temera*. The two resultant mixed matrices (see Supplemental material, File 4 [CH coding] and File 5 [DS coding]) include a total of 42 taxa and 14,108 characters. Codon positions were set *per* Aschliman *et al*. (2012b), and the matrix was run in MrBayes for 5 million generations, where variable rates were applied to molecular data as invgamma and to morphological data as gamma (Huelsenbeck & Ronquist 2001). We calculated the clade credibility, which reflects the proportion of trees in the posterior probability sample that share a given node. Parameters are pasted at the end of the Supplemental material, Files 4 and 5, to execute automatically in MrBayes.

## Institutional abbreviations

**MB.F,** Museum für Naturkunde, Berlin; **MCSNV,** Museo Civico di Storia Naturale, Verona; **MCZ,** Museum of Comparative Zoology, Harvard University, Cambridge; **MGGC,** Museo Geologico Giovanni Capellini, Bologna; **MGP-PD,** Museo di Geologia e Paleontologia dell’Università degli Studi di Padova; **MNHN,** Muséum National d’Histoire Naturelle, Paris; **NHMUK PV,** Natural History Museum, London, UK; **USNM,** National Museum of Natural History, Smithsonian Institution, Washington, DC.

## Comparative material examined

*Platyrhina sinensis*: MNHN IC.0000.1307, USNM 51295, USNM 86920, USNM 192562; *Platyrhina* sp.: USNM 130600; *Platyrhinoidis triseriata*: MCZ S749, MCZ S750, MCZ S876, MCZ S895, MCZ 99000, USNM 222020, USNM 26893, USNM 395425, USNM RAD109877; *Rhina ancyostoma*: USNM 207005; †*Tingitanius tenuimandibulus*: NHMUK PV P66857; *Zanobatus schoenleinii*: USNM 193743, USNM 193991.

## Systematic palaeontology

Class **Chondrichthyes** Huxley, 1880

Superorder **Batomorphii** Cappetta, 1980

Family **Platyrhinidae** Jordan, 1923

Genus **†*Eoplatyrhina*** gen. nov.

**Type species.** †*Platyrhina bolcensis* Heckel, 1851.

### 

#### 

##### Diagnosis

Platyrhinid characterized by the following combination of characters: rostral cartilage very long, almost reaching the anterior border of the disc; anterior fontanel extending through the entire length of the rostral cartilage with a closed and concave posterior border; nasal capsules at right angles to the rostrum; single small horn on nasal capsule; large space between the hyomandibulae and mandibular arch; approximately 132 vertebral centra (20–24 trunk centra; 113–118 centra from puboischiadic bar to the tip of tail); 15 or 16 rib pairs; 81–87 pectoral radials (35–38 propterygial, 8–10 mesopterygial, 38–41 metapterygial); 18–21 pelvic radials; short, straight and stout claspers (about 10% of total length; TL, hereafter); 20–25 caudal-fin radials on both ventral and dorsal sides (40–50 in total); thorns absent.

##### Derivation of name

The name is derived from the Greek *Ēōs*, pertaining to the sunrise, as well as to the goddess of dawn and the epoch from which the taxon is found, plus *Platyrhina*, a living thornback ray, therefore indicating a close relationship of this latter genus with the new taxon.

##### Included species

Type species only.

†***Eoplatyrhina bolcensis*** (Heckel, 1851) comb. nov.

Figs 2–7

1833–1843 *Narcopterus bolcanus* Agassiz: vol. 1: 44 (*nomen nudum*; no description or figure).

1833–1843 *Narcopterus bolcanus* Agassiz: vol. 3: 382.

1833–1843 *Narcopterus bolcanus* Agassiz: vol. 4: 38.

1835 *Narcopterus bolcanus* Agassiz: 14.

1851 *Platyrhina bolcensis* Heckel: 324 (first occurrence of name and description).

1854 *Platyrhina* (?) *bolcana*; Pictet: 277.

1860 *Platyrhina bolcensis* Heckel; Molin: 587.

1874 *Platyrhina bolcensis* Heckel; De Zigno: 177.

1894 *Platyrhina bolcensis* (Heckel) Molin; Jaekel: 106, fig. 18.

1904 *Platyrhina bolcensis* (Heckel); Eastman: 27.

1905 *Platyrhina bolcensis* (Heckel); Eastman: 351.

1922 *Platyrhina bolcensis* (Agassiz) Heckel; D’Erasmo: 12.

1980 *Platyrhina bolcensis* Heckel; Blot: 344.

1987 *Platyrhina bolcensis* Molin, 1860; Cappetta: 139.

2004 *Platyrhina bolcensis*; Carvalho: 78, fig. 12A, C.

2012 *Platyrhina bolcensis* Molin, 1860; Cappetta: 346.

2014 *Platyrhina bolcensis* Heckel, 1851; Carnevale, Bannikov, Marramà, Tyler & Zorzin: 41.

2018c ‘*Platyrhina*’ *bolcensis*; Marramà, Carnevale, Engelbrecht, Claeson, Zorzin, Fornasiero & Carnevale: 287, fig. 12C.

##### Holotype

**Figure 2.**
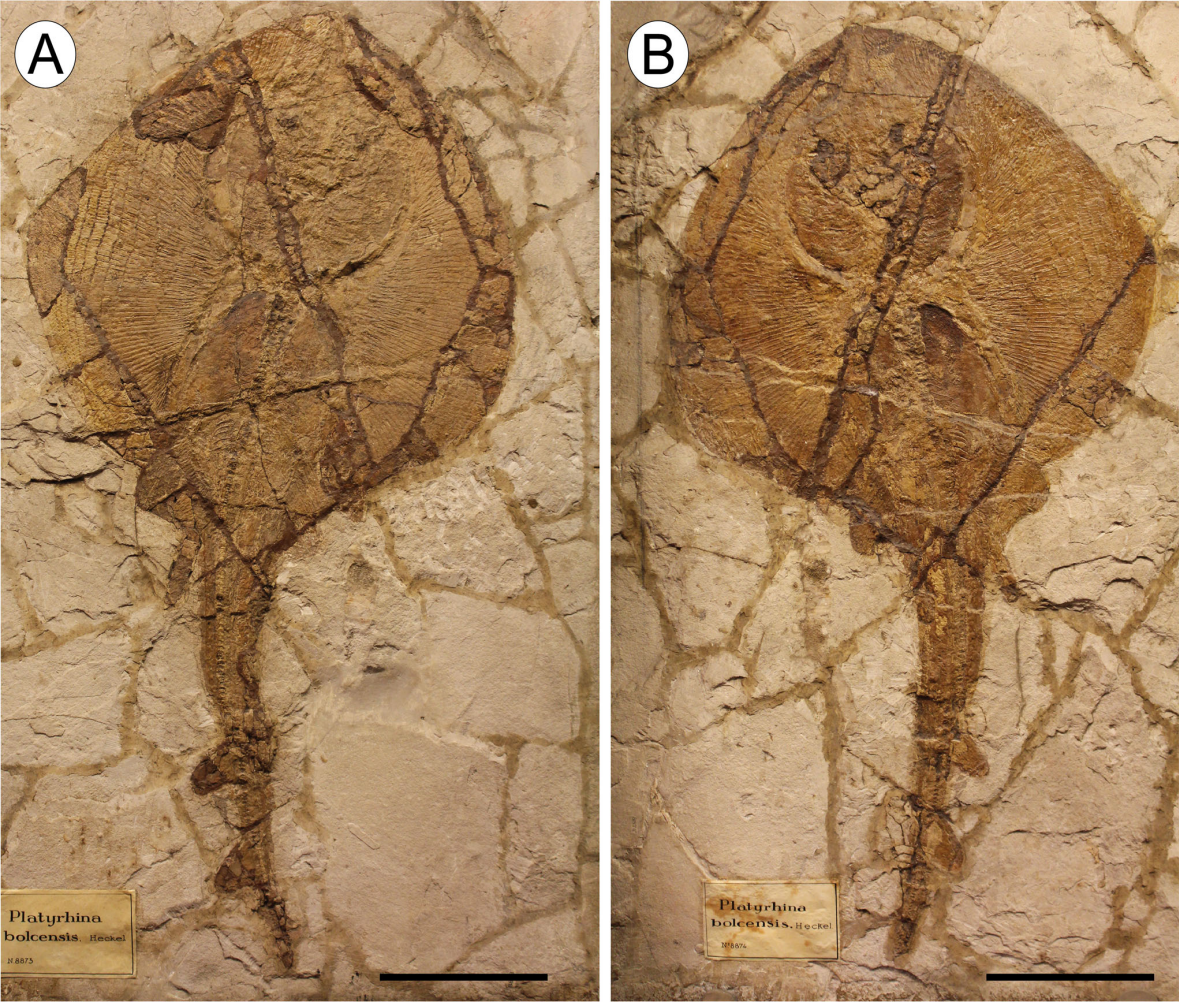
The holotype of †*Eoplatyrhina bolcensis* (Heckel, 1851) comb. nov. from the Monte Postale site in part and counterpart. **A**, MGP-PD 8873C; **B**, MGP-PD 8874C. Scale bars = 100 mm.

MGP-PD 8873C/8874C, articulated skeleton in part and counterpart, lacking the caudal fin, 338.5 mm disc width (DW, hereafter; Fig. 2).

##### Referred material

**Figure 3.**
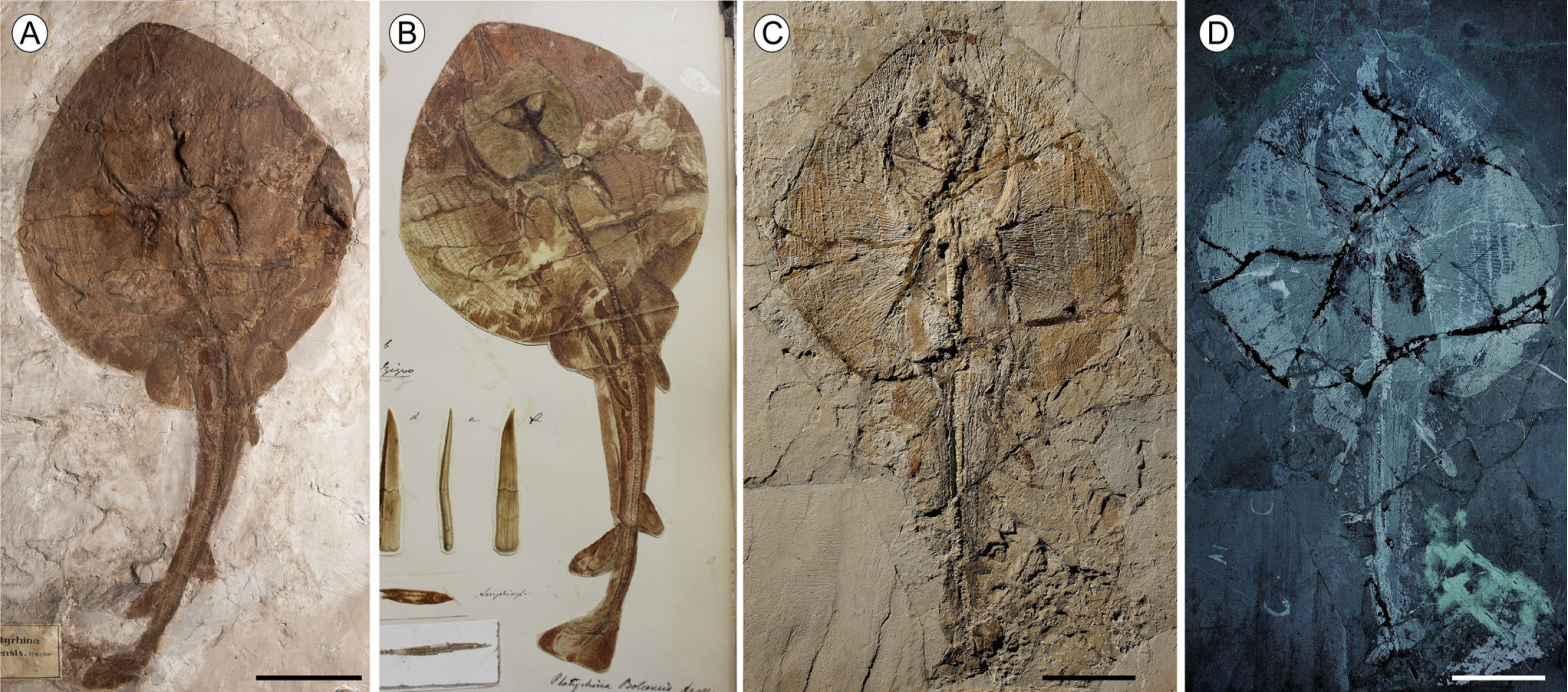
Specimens of †*Eoplatyrhina bolcensis* (Heckel, 1851) comb. nov. from the Monte Postale site. **A**, MGP-PD 26279C; **B**, original historical plate of the specimen MGP-PD 26279C, illustrated by Achille de Zigno (1813–1892). Photo: courtesy of Università degli Studi di Padova; **C**, specimen MGGC 7449; **D**, MGGC 7449 under ultraviolet light. Scale bars = 100 mm.

MGP-PD 26279C/26280C, completely articulated skeleton in part and counterpart, 384.2 mm DW, 840.3 mm TL (Fig. 3A, B); MGGC 7449/7450, articulated skeleton in part and counterpart, lacking dorsal and caudal fins, 379.4 mm DW (Fig. 3C, D).

##### Type locality and horizon

Monte Postale site, Bolca Konservat-Lagerstätte, Italy; early Eocene, Ypresian, middle Cuisian, SBZ 11 (NP 13, CNE 5); 50.7–48.9 Ma (Papazzoni *et al*. 2017).

##### Diagnosis

As for the genus.

### Description

†*Eoplatyrhina bolcensis* (Heckel, 1851) comb. nov. is represented by three partially complete articulated specimens in part and counterpart (Figs 2, 3), including the holotype (MGP-PD 8873C/8874C) and two additional specimens (MGP-PD 26279C/26280C and MGGC 7449/7450). Counts and measurements are listed in the Supplemental material (File 1, Table S1). The examined specimens are similar in size. The largest one measures 84 cm TL and 38 cm DW. The pectoral disc of †*Eoplatyrhina* gen. nov. is notably expanded, ovoid or shovel shaped, slightly longer than wide and reaching its maximum width just posterior to its mid-length. The snout is broad and rounded. The tail is not very stout, slightly longer than disc length, with two dorsal fins inserting posteriorly on the tail. The overall body shape and proportions are similar to those of the extant thornbacks *Platyrhina* and *Platyrhinoidis*.

#### 

##### Neurocranium

**Figure 4.**
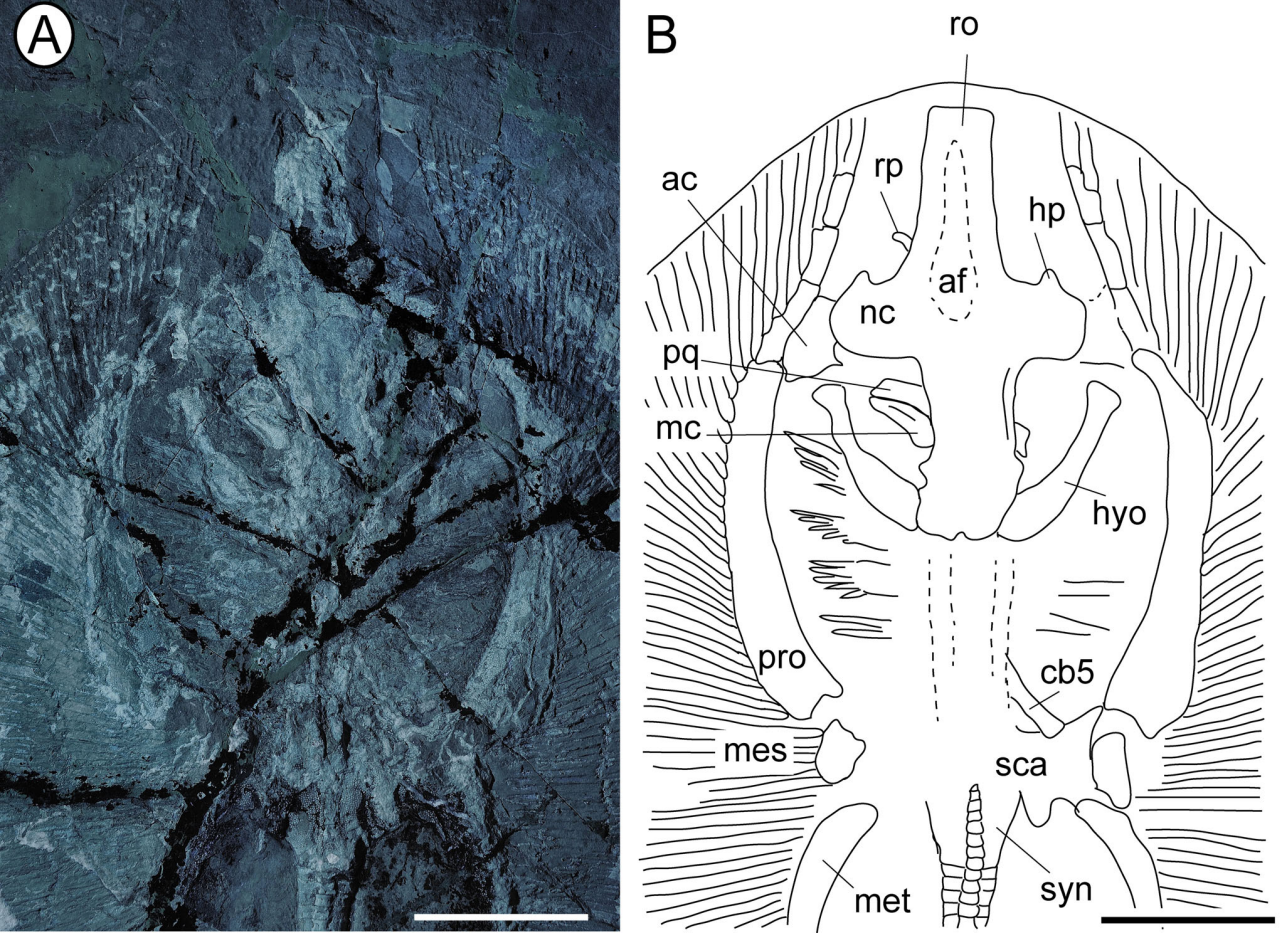
†*Eoplatyrhina bolcensis* (Heckel, 1851) comb. nov. from the Monte Postale site. **A**, MGGC 7449, close-up of the head and pectoral girdle under UV light; **B**, reconstruction. **Abbreviations: af,** anterior fontanel; **ao,** antorbital cartilage; **cb5,** fifth ceratobranchial; **hp,** horn-like process; **hyo,** hyomandibula; **mc,** Meckel’s cartilage; **mes,** mesopterygium; **met,** metapterygium; **nc,** nasal capsule; **pq,** palatoquadrate; **pro,** propterygium; **ro,** rostral cartilage; **rp,** rostral process; **sca,** scapulocoracoid; **syn,** synarcual. Scale bars = 50 mm.

**Figure 5.**
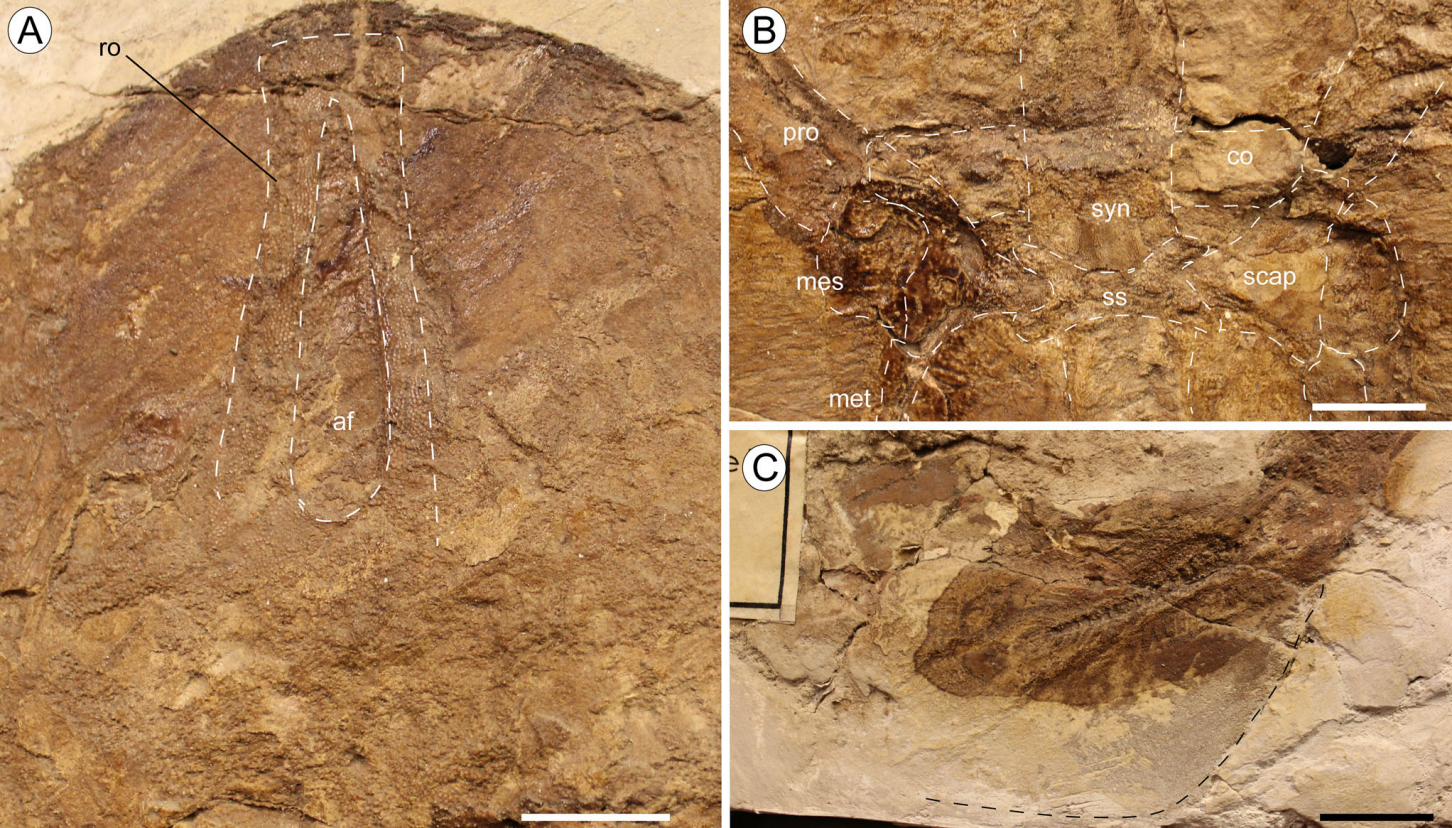
†*Eoplatyrhina bolcensis* (Heckel, 1851) comb. nov. from the Monte Postale site. **A**, MGP-PD 26279C, close-up of the rostral cartilage; **B**, close-up of the pectoral girdle (dorsal view) showing the position of the suprascapula; **C**, caudal fin of MGP-PD 26279C; the dashed line shows the original genuine outline of the fin. **Abbreviations: af,** anterior fontanel; **co,** coracoid bar of the scapulocoracoid; **mes,** mesopterygium; **met,** metapterygium; **pro,** propterygium; **ro,** rostral cartilage; **scap,** scapular process of the scapulocoracoid; **ss,** suprascapulae; **syn,** synarcual. Scale bars = 20 mm.

The rostral cartilage fails to reach the anterior margin of the disc, as in all platyrhinids. This element is long and tapers gradually anteriorly (Figs 4, 5A), resembling the condition typical of *Platyrhinoidis* and †*Tethybatis*, and differs from the short rostrum observed in †*Tingitanius* and *Platyrhina.* Unlike other platyrhinids, the anterior margin of the rostral cartilage is not pointed but trough-shaped, with the rostral node slightly expanded laterally (Figs 4, 5A). Rostral appendices at the tip of the rostrum are absent. A small rod-like process lateral to the rostral cartilage and just anterior to the nasal capsule in MGGC 7449/7450 can be interpreted as one of the two rostral processes, which are uniquely present in extant thornbacks. Although McEachran *et al*. (1996) considered these structures homologous to the rostral appendices of skates and guitarfishes, Carvalho (2004) pointed out that the rostral processes of platyrhinids, originating ventral to the rostral cartilage, might represent outgrowths of the lamina orbitonasalis, unlike the rostral appendices that are secondary chondrifications fused laterally to the rostral node. The nasal capsules are ovoid, laterally expanded, and at right angles to the rostrum, as in †*Tethybatis*. A single small horn-like process (= tab-like process of Claeson *et al*. 2013) can be recognized on the anterior margin of each nasal capsule, similar to the extant platyrhinids and †*Tingitanius*. The antorbital cartilages are well developed and plate-like and have an irregular outline. They project laterally from the postero-lateral margin of the nasal capsules and articulate distally with the propterygia. It is difficult to distinguish the preorbital process or the jugal arch, but a small and narrow postorbital process can be recognized in the otic region, just posterior to the supraorbital crest. The orbital region is longer than wide. The anterior fontanel extends through almost the entire length of the rostral cartilage and resembles an isosceles triangle with a close and concave posterior border, similar to the condition seen in †*Tingitanius*, and in contrast to the oval-shaped fontanel of *Platyrhina*, or to the figure-eight shape typical of *Platyrhinoidis*.

##### Jaws, hyoid and gill arches

Specimens of †*Eoplatyrhina bolcensis* comb. nov. are mostly preserved in dorsal view, obscuring the jaws, which are displaced and difficult to describe (Fig. 4). For the same reason, teeth are not exposed in any specimen, and therefore their morphology remains unknown. It is also unclear whether the labial cartilages are present, as in mature specimens of *Platyrhina.* The hyomandibulae are stout, robust and slightly arched, with a concave inner margin, narrow at their medial section. They project anterolaterally. As in †*Tethybatis*, there is a large space between the hyomandibulae and mandibular arch, which is interpreted by Carvalho (2004) as indicative of the presence of a large spiracular opening. In radiographs, this space is not present in *Platyrhinoidis* or *Platyrhina*, while it is present in *Zanobatus*. The distal part of the hyomandibulae appears taphonomically separated from the Meckel’s cartilage. The fifth ceratobranchials articulate with the anterior margin of the scapulocoracoid, and the remaining gill arches are poorly preserved or missing.

##### Synarcual and vertebral column

Although the synarcual can be identified as a tubular mineralized structure between the neurocranium and scapulocoracoid, its morphology remains ambiguous. The dorsally exposed specimens obscure the pattern of free centra. In †*Tingitanius*, the first exposed vertebral centrum of the synarcual is located posterior to the articulation of the suprascapular cartilage with the synarcual. In *Platyrhina*, the first free centrum is situated at the level of the scapulocoracoid articulation with the synarcual. In *Platyrhinoidis*, the first free centrum is rostral to the scapulocoracoid articulation with the synarcual. The vertebral column of †*Eoplatyrhina bolcensis* comb. nov. consists of about 132 vertebral centra, in the most complete specimen MGP-PD 26279C/26280C. There are 20–24 trunk centra (from the first distinguishable centrum to the anterior margin of the puboischiadic bar), and 113–118 from the puboischiadic bar to the tip of the tail (of these, about 23 are caudal). The vertebral centra are highly calcified, sub-rectangular in shape and anteroposteriorly compressed. There are about 15 or 16 pairs of ribs.

##### Appendicular skeleton and fins

It is difficult to describe the morphology of the coracoid bar because the specimens are mostly exposed in dorsal view, but the scapular processes of the scapulocoracoid seem to be short in MGP-PD 26279C/80C (Fig. 5B). This specimen shows a small medially fused suprascapular cartilage; this cartilage is hourglass-shaped, with concave anterior and posterior borders, exhibiting deep indentations into which the distal edges of the scapular processes of the scapulocoracoid fit. Laterally, the scapulocoracoid articulates with the proximal portion of the pterygia through equidistant condyles. The propterygium is long and arched, tapers distally and extends to the anterior disc margin (Fig. 4). The propterygium is segmented, with the first segment lying anterior to the mouth, close to the level of the antorbital cartilage. The proximal section of the propterygium does not extend far posteriorly to the procondyle, and does not articulate with the scapulocoracoid. A single unsegmented mesopterygium seems to be present. The metapterygium is as long and curved as the propterygium, but it is unclear whether it is segmented distally. The pectoral fins are clearly of the plesodic type, with radials reaching the external border of the pectoral disc. All the radials articulate with the pterygia. Each pectoral radial contains 10–12 segments and bifurcates distally only once at about the eighth segment. There are approximately 81–87 pectoral radials, of which 35–38 are propterygial, 8–10 mesopterygial, and 38–41 metapterygial. The pectoral radials of †*E. bolcensis* comb. nov. are robust, stiff and completely covered by mineralized tissue, forming the so-called ‘crustal calcification’ typical of most of batoids except the benthic stingrays and skates (Schaefer & Summers 2005).

**Figure 6.**
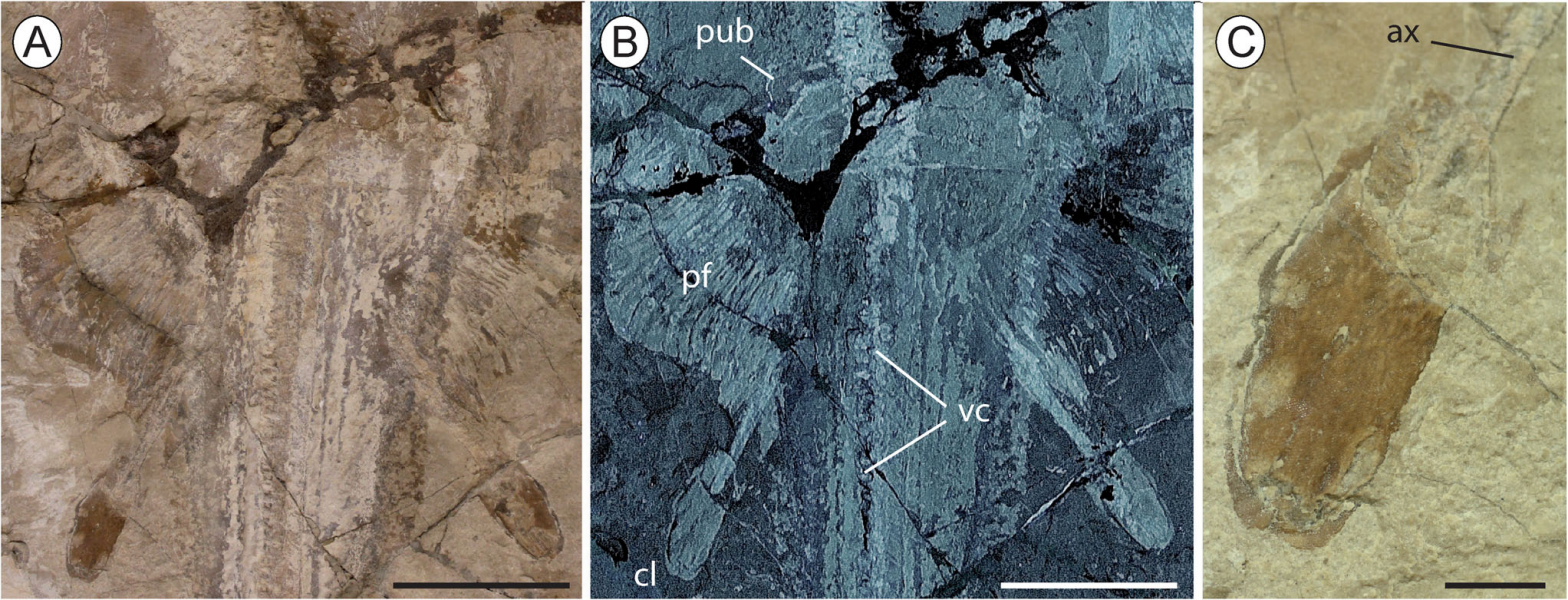
†*Eoplatyrhina bolcensis* (Heckel, 1851) comb. nov. from the Monte Postale site. **A**, close-up of the pelvic girdle and fins in MGGC 7449; **B**, the same area under ultraviolet light; **C**, detail of one of the claspers. **Abbreviations: ax,** axial cartilage; **cl,** clasper; **pf,** pelvic fin radials; **pub,** puboischiadic bar; **vc,** vertebral centra. Scale bars: A, B = 50 mm; C = 10 mm.

The puboischiadic bar is partly recognizable in MGGC 7449/7450, where it seems straight or slightly bent, narrow and plate-like (Fig. 6). It is difficult to recognize the postpelvic processes on the posterior margin of the puboischiadic bar that are typical for living platyrhinids. There are about 18–21 pelvic radials. The structure of the first pelvic radial is unclear but the pelvic condyles seem close together and not separated as in skates. All the specimens show straight and stout claspers, whose length represents about 10% TL (Fig. 6). As in *Platyrhinoidis*, their distal extremity does not reach the origin of the first dorsal fin; they differ from those characteristic of *Platyrhina*, whose clasper tips can extend beyond the first dorsal-fin origin (e.g. Last *et al.* 2016; White & Last 2016). The clasper glands are almost entirely covered by dermal denticles, and consequently their skeletal morphology is difficult to describe. However, the axial cartilage is rod-like, possibly calcified over most of its length, and extends and inserts over the complete length of the clasper to the ventral terminal cartilage.

##### Dorsal and caudal fins

†*Eoplatyrhina bolcensis* comb. nov. possesses two dorsal fins located in the posterior half of the tail. The extent of the fin radial cartilages into the fin web is not precisely ascertainable, but they are possibly aplesodic. The base of the dorsal fins has a length of about 5% TL. No impression of dorsal-fin radials is visible. The caudal fin is only preserved in MGP-PD 26279C/80C (Fig. 5C). It is about 11% TL and contains about 23 vertebrae not reaching the posterior-most border of the caudal fin. There are about 20–25 caudal-fin radials on the ventral and dorsal sides (40–50 in total), which do not reach the external margin of the caudal fin (aplesodic).

##### Dermal denticles

**Figure 7.**
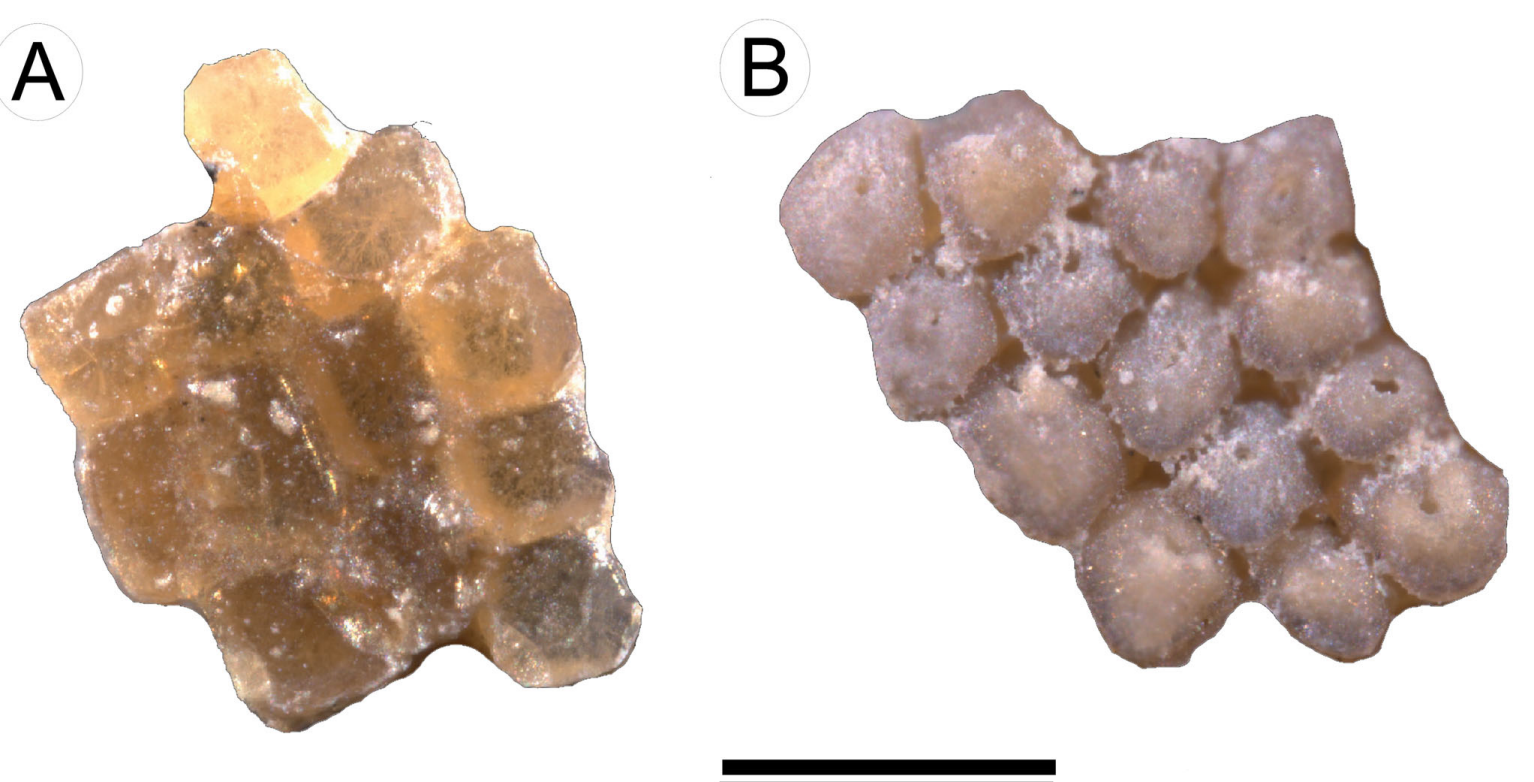
†*Eoplatyrhina bolcensis* (Heckel, 1851) comb. nov. from the Monte Postale site. **A**, dermal denticles from the tail of MGP-PD 26279C (dorsal view); **B**, dermal denticles from the tail of MGP-PD 26279C (basal view). Scale bar = 400 μm.

As in extant platyrhinids (see Deynat 2005), the entire body of †*E. bolcensis* comb. nov. is covered with numerous small dermal denticles that form a continuous and regular covering (Fig. 7). Denticle size is quite uniform across the body. Some denticles were extracted from the dorsal side of the disc of MGP-PD 26279C/80C for a detailed analysis. Their crown is about 200 μm wide and rhomboidal or lozenge-shaped (Fig. 7). The denticle root is deeper than the crown height and a nutritive foramen can be recognized near the centre. Extant thornbacks and †*Tingitanius* possess parallel rows of enlarged dermal denticles (thorns) over the posterior part of the disc and tail, a condition that was regarded as diagnostic for platyrhinids. However, this is not the case for †*Eoplatyrhina bolcensis* comb. nov. and †*Tethybatis*, in which thorns are completely absent (Carvalho 2004), possibly representing a feature supporting this sister-group relationship.

Family **Zanobatidae** Fowler, 1934

Genus †***Plesiozanobatus*** gen. nov.

##### Type species

†*Torpedo egertoni* De Zigno, 1876.

##### Diagnosis

Pectoral disc large and roughly rounded, representing 56–70% TL; tail stout and short, distinctly demarcated from the disc; two dorsal fins and caudal fin present; densely, closely set small dermal denticles forming a continuous pavement; large, rounded, scattered thorns covering the entire disc and tail; rostral cartilage absent; nasal capsules laterally expanded without horn-like processes; long propterygia extending near the anterior margin of the disc; mesopterygium absent; about 65–75 pectoral radials; puboischiadic bar narrow and moderately arched; approximately 20 pelvic-fin rays; 80–90 vertebrae; about 10 pairs of ribs.

##### Derivation of name

From the Ancient Greek word *πλησίον* (*plēsíon*) meaning ‘near’ or ‘close’, and *Zanobatus*, to remark upon its close relationship with the living panray genus.

##### Included species

Type species only.

##### Remarks

De Zigno (1876) considered that the overall similarity of the disc shape and the absence of a tail sting on the holotypic specimen MGP-PD 154Z justified the assignment of this species to the genus *Torpedo*. Later, Jaekel (1894), analysing additional, better preserved material, assigned the species †*T. egertoni* to *Platyrhina*. However, he noticed that the fossil species from Bolca might have been more closely related to *Platyrhina schoenleinii* than to *Platyrhina sinesensis* because of the general shape and proportions of the body and disc, as well as the arrangement of the pectoral radials and gill arches. *Platyrhina schoenleinii* is currently recognized as *Zanobatus schoenleinii* (see Compagno 1999).

†***Plesiozanobatus egertoni*** (De Zigno, 1876) comb. nov.

Figs 8–10

1876 *Torpedo egertoni* De Zigno: 452, pl. 17, figs 1, 2 (original occurrence of name, description and figures).

1878 *Torpedo egertoni*, De Zigno: 10, pl. 3, figs 1–2.

1894 *Platyrhina egertoni* De Zigno sp.; Jaekel: 100, pl. 2.

1904 *Platyrhina egertoni* Zigno; Eastman: 27.

1905 *Platyrhina egertoni* Zigno; Eastman: 351.

1922 *Platyrhina egertoni* (De Zigno); D’Erasmo: 12.

1980 *Platyrhina egertoni* (De Zigno); Blot: 344.

1987 *Platyrhina egertoni* (Zigno, 1876); Cappetta: 139, fig. 118A.

1991 *Platyrhina egertoni* De Zigno; Frickhinger: 204, unnumbered fig.

1991 *Torpedo* spec. ?; Frickhinger: 210, unnumbered fig.

2004 *Platyrhina egertoni*; Carvalho: 78, fig. 12B.

2012 *Platyrhina egertoni* (Zigno, 1876); Cappetta: 346, fig. 335A.

2014 *Platyrhina egertoni* De Zigno, 1878; Carnevale, Bannikov, Marramà, Tyler & Zorzin: 41.

2018c ‘*Platyrhina*’ *egertoni*; Marramà, Carnevale, Engelbrecht, Claeson, Zorzin, Fornasiero & Kriwet: 287, fig. 13A, B.

##### Holotype

**Figure 8.**
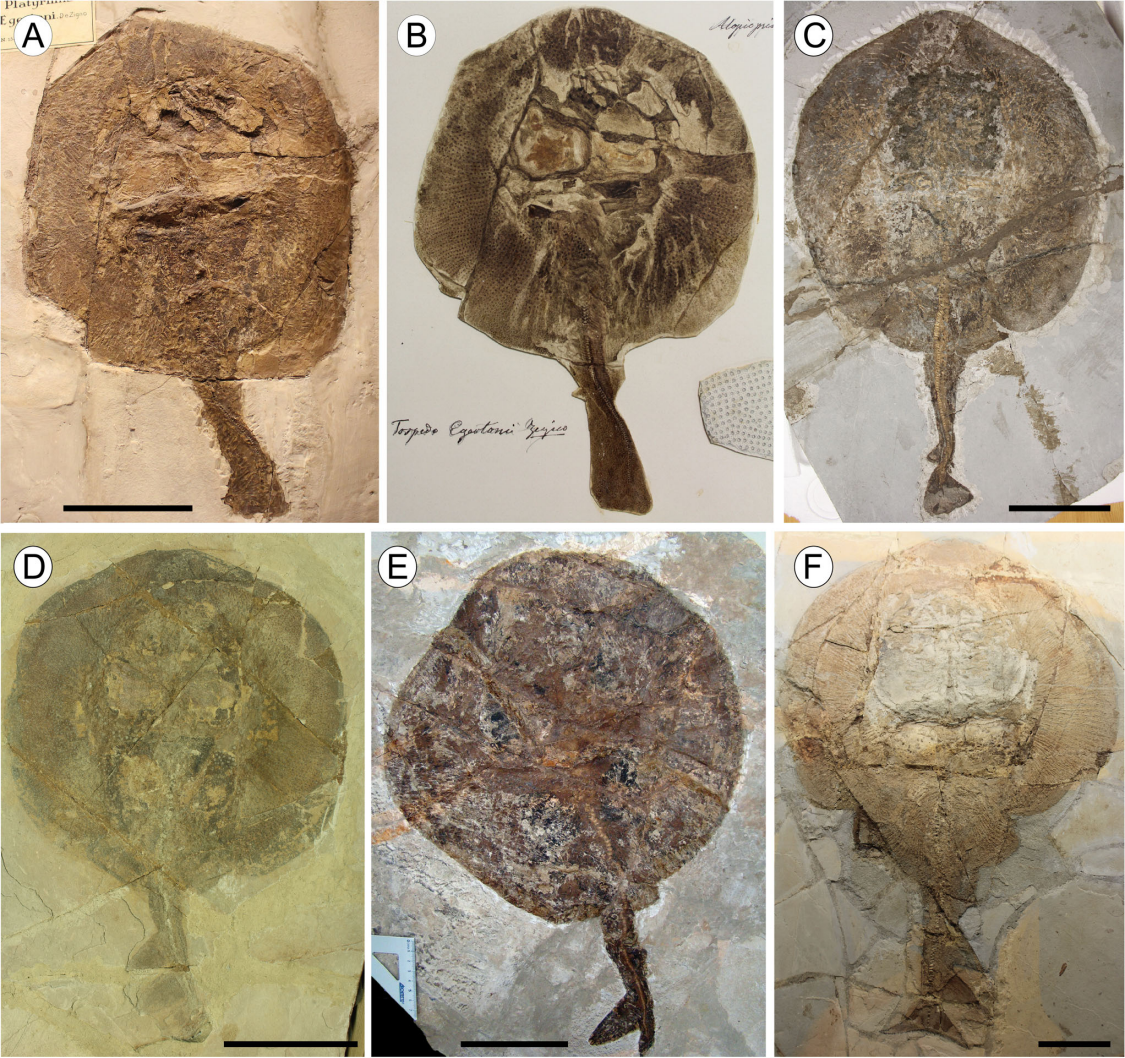
†*Plesiozanobatus egertoni* (De Zigno, 1876) comb. nov. from the Pesciara site. **A**, the holotype MGP-PD 154Z; **B**, original historical plate of the holotype illustrated and labelled as *Torpedo egertoni* by Achille de Zigno (1813–1892). Photo: courtesy of Università degli Studi di Padova; **C**, MCSNV IG.43347; **D**, MB.f 1608.1; **E**, MCSNV IG.142530; **F**, MCSNV VII.B.81. Scale bars = 100 mm.

MGP-PD 154Z, incomplete, poorly preserved articulated skeleton, 306.4 mm DW, 481.2 mm TL (Fig. 8A, B).

##### Referred material

MCSNV IG.43347, incomplete and poorly preserved articulated skeleton, 281 mm DW, 479.8 mm TL (Fig. 8C); MB.f 1608.1/2, nearly complete articulated skeleton in part and counterpart, 291.6 mm DW, 426.2 mm TL (Fig. 8D); MCSNV IG.142530, poorly preserved articulated skeleton, 336.3 mm DW, 524.5 mm TL (Fig. 8E); MCSNV VII.B.80/81, nearly complete articulated skeleton in part and counterpart, 749.2 mm DW, 1149.3 mm TL (Fig. 8F); MCSNV VII.B.88/89, partially complete articulated skeleton in part and counterpart, 311.7 mm DW, 506.3 mm TL.

##### Type locality and horizon

Pesciara site, Bolca Konservat-Lagerstätte, Italy; early Eocene, late Ypresian, middle Cuisian, SBZ 11, †*Alveolina dainelli* Zone (see Papazzoni *et al*. 2014).

##### Diagnosis

As for the genus.

### Description

**Figure 9.**
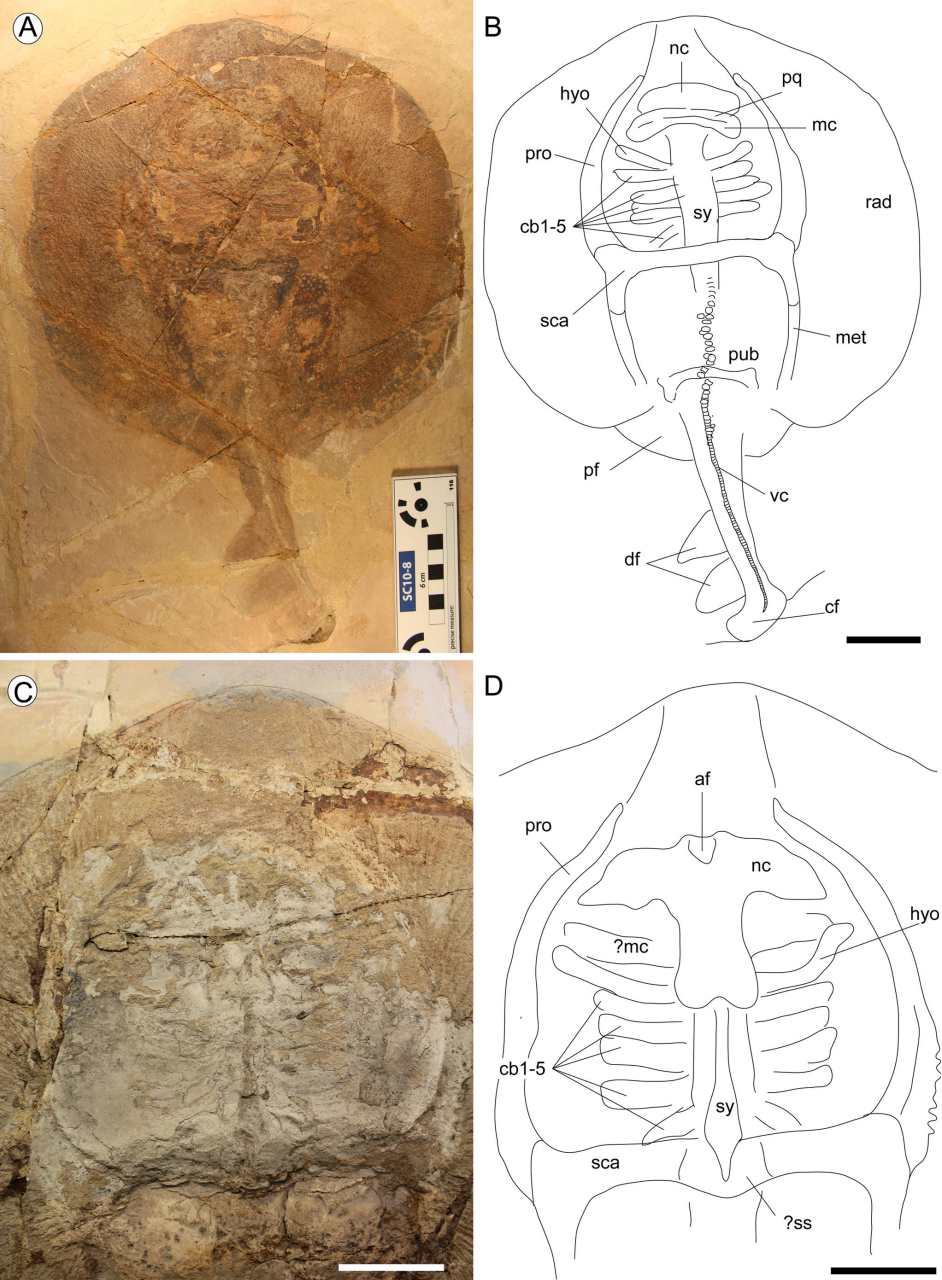
†*Plesiozanobatus egertoni* (De Zigno, 1876) comb. nov. from the Pesciara site. **A**, MB.f 1608.1; **B**, reconstruction of the body outline and main skeletal structures (denticles and radials omitted); **C**, close-up of the head and pectoral girdle of MCSNV VII.B.81; **D**, reconstruction. **Abbreviations: af,** anterior fontanel; **cb,** ceratobranchials; **cf,** caudal fin; **df,** dorsal fins; **hyo,** hyomandibula; **mc,** Meckel’s cartilage; **met,** metapterygium; **nc,** nasal capsules; **pf,** pelvic fins; **pq,** palatoquadrate; **pro,** propterygium; **pub,** puboischiadic bar; **rad,** pectoral radials; **sca,** scapulocoracoid; **sy,** synarcual; **ss,** suprascapula; **vc,** vertebral centra. Scale bars = 50 mm.

†*Plesiozanobatus egertoni* comb. nov. is represented by six specimens showing different ontogenetic stages, with the largest individual measuring more than 1 m in length (Fig. 8). Counts and measurements are shown in the Supplemental material (File 1, Table S2). The pectoral disc is large and nearly round, representing 56–70% TL. The tail is stout and short, distinctly demarcated from the disc and measuring about 40–50% TL (Fig. 9A–B). The most complete specimens show two nearly triangular dorsal fins of similar size, located well behind the pelvics; a nearly complete caudal fin is visible exclusively in MCSNV IG.43347.

**Figure 10.**
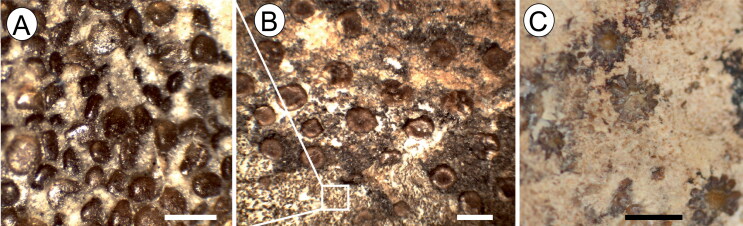
†*Plesiozanobatus egertoni* (De Zigno, 1876) comb. nov. from the Pesciara site. **A**, dermal denticles from the tail of MCSNV IG.43347; **B**, thorns from the tail of MCSNV IG.43347; **C**, thorns from the scapular region of MCSNV VII.B.80. Scale bars: A = 400 μm; B, C = 1 mm.

Although the general body shape is still detectable, a detailed analysis of all the skeletal structures is very difficult due to the generally poor preservation of the available specimens. The rostral cartilage is clearly absent in all the specimens, and a large empty space can always be recognized between the anterior propterygial radials (Fig. 9C, D). The nasal capsules are laterally expanded and do not show evidence of the horn-like processes typical of platyrhinids. The antorbital cartilages are difficult to detect but they probably articulated with the mesial margin of the propterygia. The propterygia are long, extending close to the anterior margin of the disc, well beyond the nasal capsules. The mesopterygium appears absent, as in *Zanobatus*, *Gymnura* and some pelagic stingrays, suggesting that the mesocondyle (not visible) might have been replaced by a ridge. There are about 65–75 highly calcified pectoral radials (= ‘crustal pattern’ of Schaefer & Summers 2005). Most of them articulate with the pterygia and some others articulate directly with the scapulocoracoid. The puboischiadic bar is scarcely visible in all the specimens and appears as a narrow and moderately arched bar at least in MB.f 1608.1/2. About 20 pelvic-fin rays can be recognized in the pelvic fins of †*P. egertoni* comb. nov. The most complete specimens exhibit 80–90 vertebrae and around 10 pairs of ribs. Small, imbricated and densely set dermal denticles form a continuous pavement throughout the body (Fig. 10A); their crowns are roughly rhomboid or polygonal in shape, with a flat and smooth surface. Large rounded thorns are more widely spaced, sparse and cover the whole pectoral disc and tail (Fig. 10B), whereas some scattered star-shaped thorns cover the scapular region (Fig. 10C). However, parallel antero-posteriorly directed rows of thorns are clearly absent. There are no teeth preserved in the available specimens.

## Phylogenetic analyses

### Parsimony

**Figure 11.**
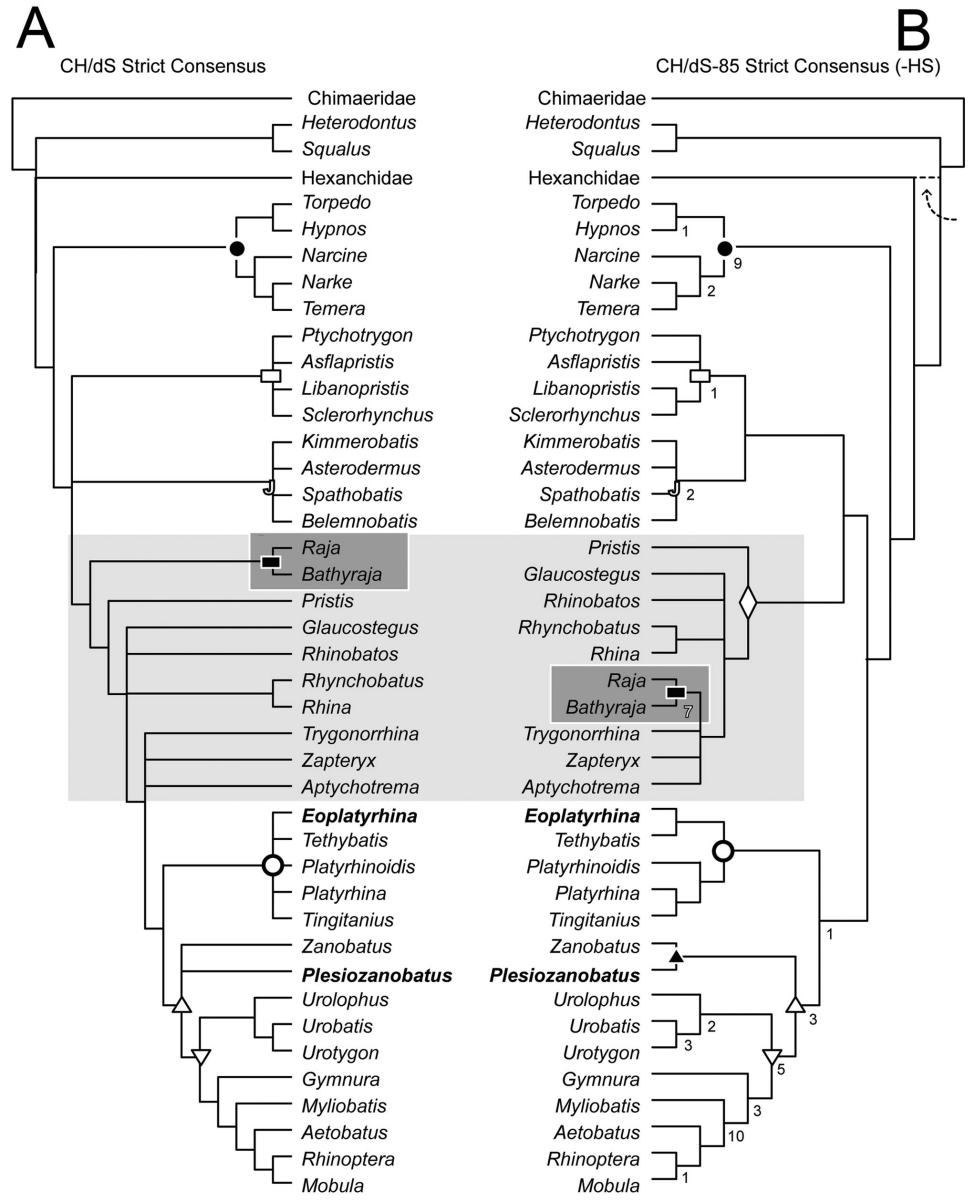
Comparative strict consensus trees from parsimony analyses run in PAUP showing the hypothetic relationships of †*Eoplatyrhina* gen. nov. and †*Plesiozanobatus* gen. nov. (in bold) among batoids. **A**, consensus tree of total dataset with suprascapular coding according to Compagno (1999) and to Da Silva *et al*. (2018); **B**, consensus tree of total dataset with suprascapular coding according to Compagno (1999) and Da Silva *et al*. (2018) with the exclusion of character 85 and excluding *Heterodontus + Squalus* (-HS). Dashed line from Hexanchidae represents a polytomy recovered in CH, compared to DS. Grey inset indicates the section of the tree with most variability, as it relates to the position of Rajidae. Numbers above branches reflect the Bremer support. Closed circles = Torpediniformes; open circles = Platyrhindae; closed triangle = Zanobatidae; open triangle = Myliobatiformes + Zanobatidae; upside-down open triangle = Myliobatiformes; closed rectangles = Rajiformes; open rectangles = Sclerorhynchoidea; open diamond = Rhinopristiformes; open J = Jurassic batoids. See Table 1 for all tree statistics and see Supplemental material, File 1, Fig. S1 for all characters mapped in support of tree B.

The tree statistics for the phylogenetic analysis of morphological data performed using PAUP are available in Table 1, and consensus tree topologies are compared in Figure 11. The consensus tree topological hypotheses recovered are identical with respect to the matrix coding of the suprascapulae according to Compagno (1999; CH coding, Fig. 11A) and the coding of Da Silva *et al.* (2018, DS coding, Fig. 11A), though tree scores are different. The hypotheses are also identical (except for the placement of Hexanchidae), and much better resolved with the exclusion of character 85, which refers to the shape of the second hypobranchial (Fig. 11B). Character mapping is provided on the tree topology of Figure 11B and in the Supplemental material (File 1, Fig. S1). We also performed an analysis where *Heterodontus* and *Squalus* were excluded, following a reviewer’s comments regarding the outgroups included and their coding. Of note, there is no difference with respect to the ingroup hypothesis recovered in Figure 11B.

**Table 1.** Tree statistics for parsimony analyses. **Abbreviations: CH,** coding follows Compagno (1999); **CI,** consistency index; **DS,** coding follows Da Silva *et al.* (2018); **RI,** retention index. In CH-85 and DS-85 analyses were run excluding the character 85. In CH-HetSqua and DS-HetSqua the taxa *Heterodontus* and *Squalus* were excluded from -85 nexus files.

Analysis	Tree #	Steps	CI	RI	Consensus Tree
CH total	144	231	0.5801	0.8574	Fig. 11A
DS total	72	229	0.5808	0.8590	Fig. 11A
CH-85	16	226	0.5796	0.8563	Fig. 11B
dS-85	8	225	0.5778	0.8561	Fig. 11B
CH -HetSqua	8	222	0.5856	08526	Fig. 11B
DS -HetSqua	8	222	0.5856	0.8526	Fig. 11B

**Figure 12.**
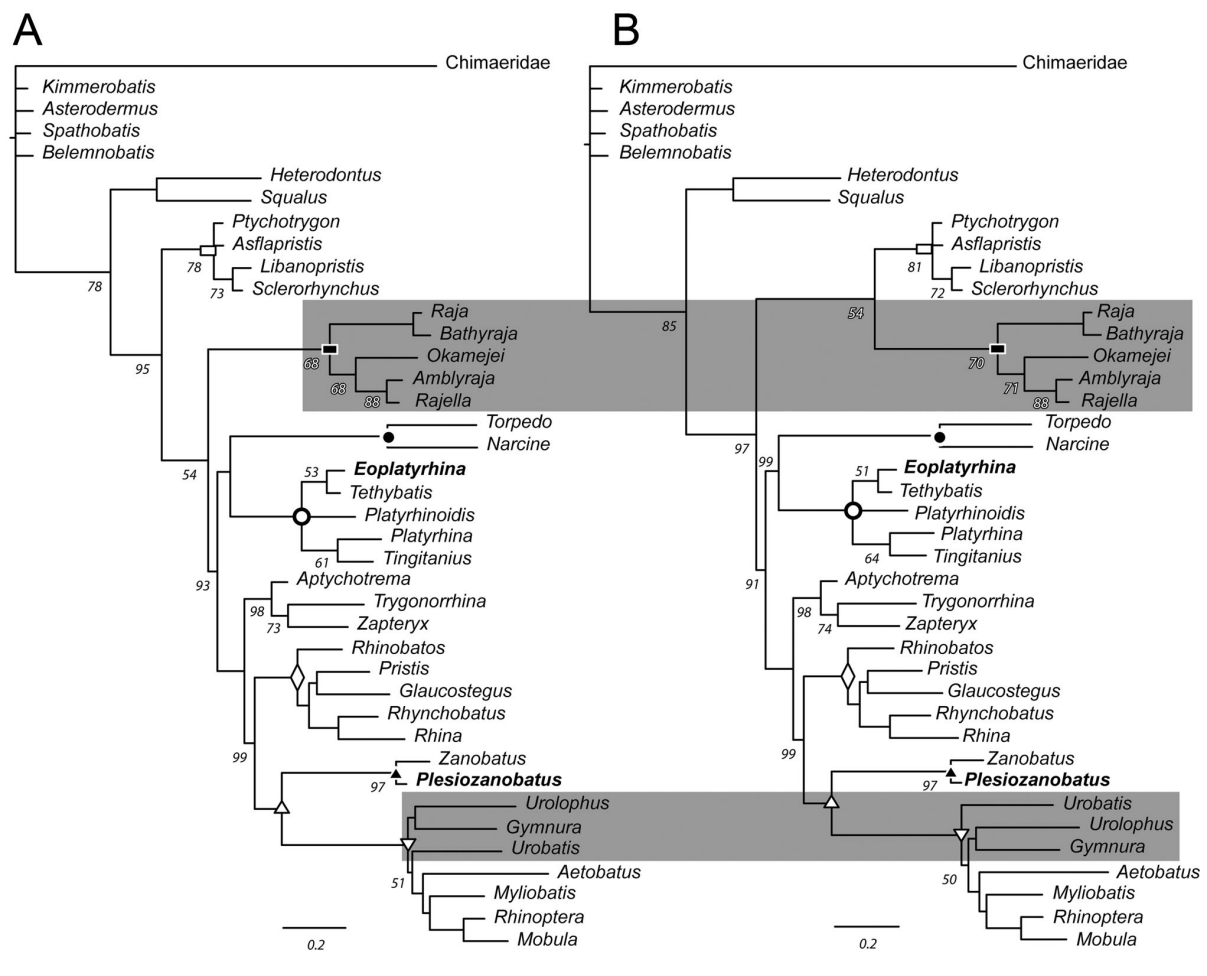
Phylogram recovered under Bayesian analyses of the total evidence data sets. **A**, hypothesis based on coding the suprascapula according to Compagno (1999); **B**, grey inset reflects how the hypothesis of relationships differs from the tree presented in A when derived from a matrix coding the suprascapula according to Da Silva *et al*. (2018). Numbers at branches reflect clade credibility. Clade credibility = 100 for branches lacking numbers. Closed circles = Torpediniformes; open circles = Platyrhindae; closed triangle = Zanobatidae; open triangle = Myliobatiformes + Zanobatidae; upside-down open triangle = Myliobatiformes; closed rectangles = Rajiformes; open rectangles = Sclerorhynchoidea; open diamond = Rhinopristiformes.

As the morphological matrix was primarily modified from Villalobos-Segura *et al*. (2019), we make comparisons to figure 12 of that study. Major clades of Batoidea are all recovered, including Torpediniformes, Jurassic batoids, sclerorhynchoid taxa, Rhinopristiformes (*sensu* Last *et al*. 2016; recovered when character 85 is excluded), Rajidae (*Raja + Bathyraja*), Platyrhinidae, and Myliobatiformes. However, the relationships among these major clades differ from the hypothesis of Villalobos-Segura *et al*. (2019). To begin with, the outgroup to all remaining batoids is the Torpediniformes, not the Jurassic batoids (see Fig. 11). In our analysis that included character 85, the Jurassic batoids, sclerorhynchoids and remaining batoids form a polytomy (Fig. 11A). With character 85 excluded, Jurassic batoids and sclerorynchoids are each other’s closest sister taxa, together forming a sister relationship to Rhinopristiformes. Rajidae (*Raja + Bathyraja*) are nested among Rhinopristiformes. Rajiformes as defined by Villalobos-Segura *et al*. (2019) was a clade of extant skates, sister taxon to the extinct clade of sclerorhyncoid batoids, with the latter being clearly separated from the phenetically similar sawfishes (e.g. *Pristis*). This relationship is not recovered by our parsimony analyses. As recovered in Villalobos-Segura *et al*. (2019), Platyrhinidae are sister taxon to a clade including Myliobatiformes + Zanobatidae.

In this section, we describe the parsimony hypotheses recovered with the exclusion of character 85 in our study (Fig. 11B and Supplemental material, File 1, Fig. S1). Batoidea are supported to the exclusion of their outgroups by 10 unambiguous character transformations. Among Batoidea, Torpediniformes form a monophyletic clade with 14 unambiguous character transformations, which is resolved as sister taxon to all remaining batoids. The clade of remaining batoids is supported by five unambiguous character transformations.

The clade of Jurassic + sclerorhynchoid batoids is supported by two unambiguous character transformations: calcified suprascapulae are absent (ch. 5[1 → 0]) and a preorbital process is absent (ch. 33[0 → 1]). Rhinopristiformes is supported by three unambiguous character transformations: a scapulocoracoid that is elongate between the mesocondyle and metacondyle (ch. 56[0 → 1]), some pectoral-fin radials that articulate directly with the scapulocoracoid (ch. 60[0 → 1]), and the presence of differentiated lateral uvulae on teeth (ch. 83[0 → 1]). These three clades form the sister taxon to the clade Platyrhinidae + (Myliobatiformes + Zanobatidae), which is supported by five unambiguous character transformations: pectoral propterygia that extend towards the anterior aspect of the disc (ch. 57[0 → 1]) – specifically, a distal propterygium that reaches beyond the nasal capsules (ch. 93[0 → 2]) – as well as pectoral radials that also reach beyond the nasal capsules (ch. 94[0 → 2]). Additionally, the clade including Platyrhinidae, Myliobatiformes and Zanobatidae is supported by anterior nasal lobes that are moderately expanded medially to cover most of the medial half of the naris and onto the internarial space (ch. 95[0 → 1]), and a diagonal coracohyoideus muscle (ch. 103[0 → 2]).

Most pertinent to our study is the position of the Bolca fossils traditionally considered members of the thornback ray family Platyrhinidae. The family forms here a monophyletic clade, sister to the grouping formed by Zanobatidae + Myliobatiformes in both CH and DS analyses (Fig. 11A, B). This arrangement is consistent with the results of McEachran *et al*. (1996) and Aschliman *et al*. (2012a), but contrasts with the most recent molecular studies that place the platyrhinids as sister to the electric ray order Torpediniformes (Naylor *et al*. 2012; Bertozzi *et al*. 2016; Last *et al*. 2016). These differences between molecular and morphological analyses are justified by the absence of unambiguous morphological synapomorphies shared by Torpediniformes and Platyrhinidae (see also Villalobos-Segura *et al*. 2019). The relationship of platyrhinids and zanobatids forming successive sister taxa to myliobatiforms, detected in our study, also contrasts with the recent morphological analysis of Brito *et al.* (2019) who recovered the clade Platyrhinidae + †*Britobatos* as the sister group of the node formed by the clade †*Stahlraja* + (†*Tlalocbatos* + (*Aptychotrema* + *Zapteryx* + *Trygonorrhina*)), with this relationship supported by two homoplastic characters: pectoral radials extending far beyond the nasal capsules, and scapulocoracoid elongated between mesocondyle and metacondyle (ch. 34[2] and ch. 43[1] of Brito *et al*. 2019). However, in our study these two features appear independently derived for platyrhinids and trygonorrhinids.

In our analyses, the monophyly of Platyrhinidae is supported by the presence of two unambiguous character transformations: rostral processes (ch. 30[0 → 1]; consistency index [CI] = 1.00), and horn-like processes on the anterior margin of nasal capsules (ch. 79[0 → 1]). The presence of well-developed antorbital cartilages, variously shaped and with irregular outline (ch. 9[1]), has been used by Villalobos-Segura *et al*. (2019) to provide a shared feature between platyrhinids and electric rays. However, in our analysis this feature appears independently derived for the two clades. †*Eoplatyrhina* gen. nov. is recovered as a genuine thornback ray that is sister to †*Tethybatis*. They share the absence of thorns (ch. 97[0]). †*Tethybatis* is distinguished from †*Eoplatyrhina* in possessing long claspers (ch. 67[0 → 1]. *Platyrhinoidis* is recovered as sister to †*Tingitanius* + *Platyrhina*, supported by the presence of parallel rows of enlarged denticles (ch. 80[1]; CI = 1.00). †*Tingitanius* + *Platyrhina* are distinguished from *Platyrhinoidis* in possessing a pair of long claspers (ch. 67[1]). Furthermore, the position of the first enclosed vertebral centrum within the synarcual of *Platyrhinoidis* is at the level of the suprascapular articulation with the synarcual, rather than posterior to it (ch. 78[2 → 1]). This placement of †*Tingitanius* contrasts with the results of Claeson *et al*. (2013) who recovered †*Tingitanius* as sister to *Platyrhinoidis* because of the absence of labial cartilages and incipient lateral uvulae on teeth. However, updated coding in our matrix for the absence/presence of lateral uvulae, following Villalobos-Segura *et al*. (2019), leads to a hypothesis that considers the absence of labial cartilages in *Platyrhinoidis* and †*Tingitanius* to be independently derived.

Zanobatidae is recovered as sister taxon to Myliobatiformes (Fig. 11A, B), supported by eight unambiguous character transformations: rostral cartilage absent (ch. 25[1 → 0]); presence of a hyomandibula-meckelian ligament (ch. 44[0 → 1]); a mesocondyle replaced with a ridge (ch. 56[0 → 3]); proximal section of the propterygium extending behind the procondyle (ch. 59[0 → 11]); narrow and moderately to strongly arched puboischiadic bar without distinct lateral processes (ch. 64[0 → 1]); dorsal margin clasper cartilages with medial flange (ch. 68[0 → 1]); a unique condition of the ventral terminal cartilages, which are folded ventrally along their long axis to form a convex flange (ch. 69[0 → 2]; CI = 1.00); and a ball-and-socket articulation between the suprascapula and scapulocoracoid (ch. 82[1 → 3]). Our detection of panrays as sister to the stingrays is consistent with the morphological and molecular analyses of Aschliman *et al*. (2012a), Bertozzi *et al*. (2016) and Last *et al*. (2016). Conversely, Naylor *et al*. (2012) recovered *Zanobatus* as a genuine member of the Rhinopristiformes, although the authors pointed out that this placement was model-dependent for that dataset.

Within Zanobatidae, †*Plesiozanobatus* gen. nov. is recovered as sister to *Zanobatus* and is distinguished from Myliobatiformes by possessing pectoral radials that directly articulate with the ridge replacing the mesopterygoid (ch. 60[0 → 1]) (see McEachran *et al*. 1996, fig. 9C). A similar condition where pectoral radials are directly articulated with the scapulocorocoid has been derived independently from the Rhinopristiformes. Myliobatiformes is distinguished from Zanobatidae by 14 unambiguous character transformations, mapped in Supplemental material, File 1, Fig. S1.

### Bayesian analysis

All major clades of batoids are recovered in the total evidence analyses that accounted for alternate codings of the suprascapular cartilages in outgroup taxa (CH, DS) and excluded the developmentally variable character of the second hypobranchial. Jurassic batoids are recovered in a polytomy among outgroup Chimaeridae and crown Neoselachii (Fig. 12). The position of Rajidae is the primary difference between the DS and CH hypotheses of batoid relationships.

The ultimate structure of the Bayesian phylogram from the CH analysis resembles that published by Aschliman *et al*. (2012b), in that among crown Batoidea, Rajiformes is the sister taxon to remaining crown batoids (clade credibility CH = 54; Fig. 12A). The alternative hypothesis has weak support for a monophyletic clade of Rajiformes (clade credibility DS = 54; Fig. 12B) that includes the extinct sclerorhynchoid batoids. When using CH and DS coding, †*Sclerorhynchus* and †*Libanopristis* are sister taxa and in a polytomy with †*Ptychotrygon +* †*Aslflapristis* (Fig. 12A, B). Sclerorhynchoids, whether in a sister taxon relationship with Rajidae or not, are the sister taxon to the remaining batoids.

Also resembling the Aschliman *et al.* (2012b) hypothesis, the position of Torpediniformes as the sister taxon to Platyrhinidae is present in both analyses. Rhinopristiformes is paraphyletic. The ‘guitarfish-1’ group of Aschliman *et al*. (2012b) now includes *Aptychotrema* as the sister taxon to *Trygonorrhina* + *Zapteryx*, as in the morphological hypothesis. In the morphological hypothesis, ‘guitarfish-1’ is highly nested within the Rhinopristiformes and sister to *Rhinobatos*. The ‘guitarfish-2’ group is identical to that of Aschliman *et al.* (2012b). The position of Rajiformes (Fig. 12B) contrasts with that of Bertozzi *et al*. (2016), who recover Torpediniformes as the sister taxon to all remaining crown Batoidea. Within Myliobatiformes, the relative position of *Urobatis* changes based on CH/DS coding from sister to Myliobatidae or sister to *Urolopus + Gymnura*, respectively, with CH coding more similar to the Aschliman *et al.* (2012b) hypothesis.

As it pertains to the new fossils from Bolca, Platyrhinidae is predicted with a clade credibility of 100 and, as in the parsimony hypothesis, †*Eoplatyrhina* gen. nov. is sister taxon to †*Tethybatis* (clade credibility CH = 53; clade credibility DS = 51), *Platyrhina* is sister taxon to †*Tingitanius* (clade credibility CH = 61; clade credibility DS = 64) and *Platyrhinoidis* is unresolved relative to the other thornback rays. †*Plesiozanobatus* is sister taxon to *Zanobatus* with a clade credibility of 97 in both analyses, and together they are sister taxon to a monophyletic Myliobatiformes (clade credibility = 100).

## Discussion

### Notes on †‘*Platyrhina*’ *gigantea* (Blainville, 1818)

**Figure 13.**
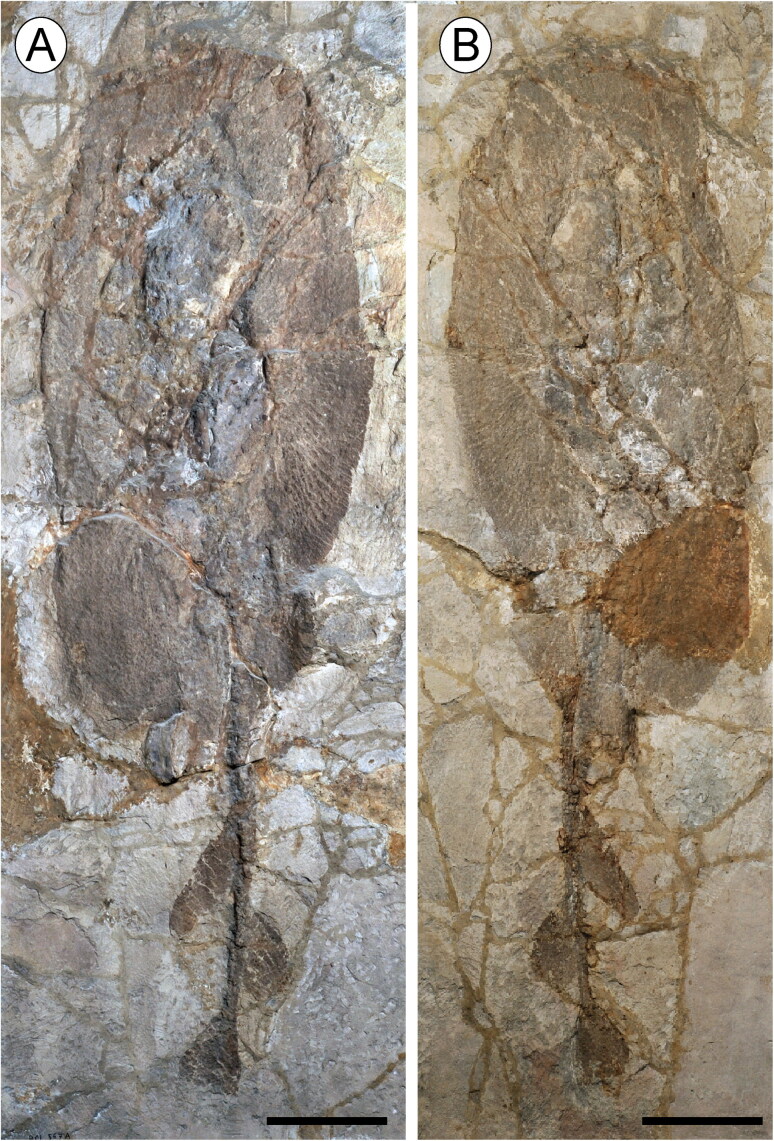
†‘*Platyrhina*’ *gigantea* (Blainville, 1818), MNHN F.Bol567, in **A**, part and **B**, counterpart from the Pesciara site. Scale bars = 100 mm.

A single specimen in part and counterpart (MNHN F.Bol567) housed in the Muséum National d’Histoire Naturelle, Paris (Fig. 13) was figured and assigned by Volta (1796, pl. 61) to *Raja torpedo*, which is currently a junior synonym of *Torpedo torpedo* (Linnaeus, 1758). Blainville (1818), without further description or figure, created a new species (†*Narcobatus giganteus*) based on that specimen (*Narcobatus* is a junior synonym of *Torpedo*), whereas Molin (1860) assigned it to the genus *Narcine*. De Zigno (1874) reported another specimen housed in MCSNV, whose measurements may correspond to those of MCSNV VII.B.80/81 (assigned herein to †*Plesiozanobatus* gen. nov.; Fig. 8F), and described it as *Torpedo gigantea* (labelled in the MCSNV as †*Platyrhina gigantea*, authors’ pers. obs.). Jaekel (1894) was unable to locate the specimen figured by Volta (1796) and, solely based on the poorly detailed drawing provided by Volta, concluded that the species should have been assigned to *Platyrhina* (see Eastman 1904, 1905). The anatomical analysis of specimen MNHN F.Bol567 is extremely problematic, it being a specimen preserved in a heavy limestone slab more than 2 m long and mounted very high on a wall at the MNHN. A cursory analysis of this badly preserved and possibly deformed specimen detected a short but slender tail, two dorsal fins and a caudal fin, but the pectoral disc was unlikely to have been anteroposteriorly elongated. No cranial or postcranial structures are recognizable. In addition, the specimen seems to have been erroneously assembled, and possibly painted, making it very difficult to interpret reliable diagnostic characters and thereby preventing a possible assignment to any known batoid taxon or group. Due to the extremely problematic taxonomic interpretation of this specimen, we therefore suggest †*Platyrhina gigantea* (Blainville, 1818) be considered a *nomen dubium*.

### Comparison and relationships

The monophyly of the family Platyrhinidae has been defined by the presence of rostral processes, postpelvic processes on the puboischiadic bar, plate-like irregularly-shaped antorbital cartilages, and the rostral cartilage failing to reach the tip of the snout (Carvalho 2004; McEachran & Aschliman 2004; Aschliman *et al*. 2012a; Claeson *et al*. 2013). As such, the analysis of the skeletal morphology of †*Eoplatyrhina bolcensis* comb. nov. revealed the presence of several features that support the inclusion of this taxon within the family Platyrhinidae, with strong support in the Bayesian analyses (clade credibility, 100). †*Eoplatyrhina* gen. nov. can be distinguished from the other members of the family (Supplemental material, File 1, Table S3) by the presence of a long rostral cartilage (very short in *Platyrhina*), a triangular anterior fontanel (oval in *Platyrhina* or figure-eight-shaped in *Platyrhinoidis*), nasal capsules at right angles to the rostrum (anteriorly directed in *Platyrhina* and *Platyrhinoidis*), a small horn on nasal capsules (possibly absent in †*Tethybatis*), a large space between the hyomandibulae and mandibular arch (small in living taxa), and thorns absent (present in living platyrhinids and †*Tingitanius*). The vertebral column of †*Eoplatyrhina* gen. nov. consists of about 132 vertebral centra and 15–16 pairs of ribs, whereas *Platyrhinoidis* and †*Tethybatis* are characterized by fewer vertebrae and fewer pairs of ribs (Supplemental material, File 1, Table S3). The number of pectoral radials in †*Eoplatyrhina* gen. nov. is higher than in all the extinct and living platyrhinids, whereas its claspers are short, with the distal extremity failing to reach the first dorsal fin, unlike the very long claspers characteristic of *Platyrhina* and †*Tingitanius*. Low clade credibility among the interrelationships of Platyrhinidae, in particular the fragility of the relationships of the Cretaceous taxon †*Tingitanius* among Platyrhinidae based on recoding single character states, may reflect the limited number of currently identified apomorphies of extinct and extant taxa. Despite this limited number of apomorphies, we recover a sister-taxon relationship between the Eocene thornback rays †*Eoplatyrhina* + †*Tethybatis.*

Based on our analyses, Zanobatidae is unambiguously recovered as sister taxon to Myliobatiformes, using parsimony and Bayesian inferences (Figs 11, 12). Zanobatidae is no longer monotypic, now defined as *Zanobatus* + †*Plesiozanobatus egertoni* comb. nov. with a clade credibility of 97 and one unambiguous character transformation of certain pectoral fin radials articulating directly with the ridge replacing the mesopterygia (ch. 60[0 → 1]). A similar condition, where pectoral radials are directly articulated with the scapulocorocoid, is derived independently in the Rhinopristiformes. We consider this character to warrant thorough reexamination and worthy of a developmental study to further distinguish these morphologies. The extant panray *Zanobatus* includes two species (*Z. schoenleinii* and *Z*. *maculatus*) whose meristic features and bodily proportions (Séret 2016) are considerably different from those of †*Plesiozanobatus* gen. nov. (see Supplemental material, File 1, Table S3). We therefore consider †*Plesiozanobatus* gen. nov. to be unambiguously sister to the extant *Zanobatus*.

### Bayesian notes and outgroup impact

The CH parsimony analysis for the morphological data set that included Chimeridae, Hexanchidae, *Heterodontus* and *Squalus* resulted in 16 most parsimonious trees when character 85 is excluded. Eight most parsimonious trees resulted for the DS analysis when character 85 is excluded, the consensus trees of which (Fig. 11B) recovered the Torpediniformes as the sister taxon to all other batoids, and Platyrhinidae and Zanobatidae forming successive sister taxa to Myliobatiformes, as in Aschliman *et al*. (2012a). Unlike Aschliman *et al*. (2012a), there was more resolution among ‘guitarfish’ groups, in congruence with the first morphological hypothesis presented here, i.e. Rajiformes and Rhinopristiformes are recovered as monophyletic. The most novel aspect of these hypotheses is the relationship between the sclerorhynchoid batoids and a monophyletic Jurassic batoid clade, which are paraphyetic with crown batoids (CH) or sister to each other (DS). The Bayesian analysis recovers Jurassic batoids as outside Euselachia.

During the first iteration of the Bayesian analysis for this study, only sequence data for *Heterodontus* were added, and Hexanchidae was excluded because we had too few molecular data for this taxon. The results of that study, however, predicted that *Heterodontus* was nested among the extinct Rajiformes, i.e. sclerorhyncoid sawfishes. This seemed flawed considering we had yet to score the morphology for *Heterodontus* and could not obtain sequence data for the extinct sclerorhyncoid sawfishes. Thus, we added the morphological data for *Heterodontus* to the matrix and also included total evidence for *Squalus*. The ultimate hypothesis resulted in a sister-group relationship among *Heterodontus* + *Squalus* and a monophyletic crown Batoidea. This remained the case after several iterations and variations of coding among outgroup and ingroup taxa, as per suggestions by an anonymous reviewer (Table 1; Figs 11, 12).

Furthermore, there were several characters warranting additional scrutiny. Namely, there are differing interpretations about the pectoral morphology and branchial morphology in elasmobranchs that might impact character transformations and interpretations of phylogeny. We therefore prepared six variations of character coding and outgroup taxa included (Table 1). The variations of the morphological matrix were with regard to character 5, the presence of a scapulocoracoid. Compagno (1999) considers the scapular process to be the unsegmented dorsomedial projection from the scapulocorocoid. Articulating with the scapular process might be another small cartilage, the suprascapula. In sharks, we considered the segmented distal portion off the scapular process to be a suprascapula, as in the case of *Squalus*. In Da Silva *et al.* (2018, figs 1A, 3B), scapular morphology is discussed for Squaliformes, where the projection from the scapulocorocoid is defined as the ‘scapula’ in sharks (e.g. *Squalus* and *Heterodontus*) with a segmented ‘scapular process’, while in batoids, a non-segmented projection is the scapular process. This segmental scapular process is what we considered to be the suprascapula of Compagno (1999). We coded Compagno (CH) and Da Silva (DS) independently; the result was reasonably well-resolved consensus trees among major clades of batoids with the CH and DS codings being nearly identical to each other, with the exception of the position of outgroups in a polytomy (Fig. 11). Further, there was a great deal of resolution when character 85 was excluded (Fig. 11B). We also ran parsimony analyses excluding *Heterodontus* and *Squalus* as outgroups, as in Villalobos-Segura *et al.* (2019); this had no impact on the ingroup topology once character 85 was removed (Fig. 11B). Generally, there were no major differences in the outcomes of the ingroup relationships. With this aspect of morphology in particular – branchial element development – we note there will be a great benefit from conducting more ontogenetic studies to understand the early life stages of elasmobranchs and their usefulness for interpreting homologies among elasmobranch species.

### Palaeoecology, palaeobiogeography and evolutionary significance

The palaeoecological role of platyrhinids and zanobatids from the Bolca Lagerstätte has never been investigated. All the specimens of †*Eoplatyrhina bolcensis* comb. nov. are from the Monte Postale site. Living representatives of the Platyrhinidae are inshore batoids today represented by four species of *Platyrhina*, and a single species of *Platyrhinoidis* occurring in warm-temperate to tropical coastal marine waters of the north-western and eastern Central Pacific, mostly occurring off sandy beaches, in muddy enclosed bays, and near kelp beds and shallow mud bottoms (Compagno & Last 1999; Iwatsuki *et al*. 2011; Last *et al*. 2016). Quantitative palaeoecological and taphonomic analyses of the fish assemblage of Monte Postale suggests that the fossiliferous sediments accumulated close to an emerged coastal area characterized by mangroves and seagrass, in a coral reef context in the western Tethys (Marramà *et al*. 2016; Vescogni *et al*. 2016). From this perspective, it is reasonable to suggest that the Bolca platyrhinids inhabited the warm shallow-water habitats of the Monte Postale palaeobiotope (Marramà *et al*. 2016; Vescogni *et al*. 2016). In addition, it is interesting to note that among the coeval Tethyan and Boreal Eocene deposits, the presence of thornback rays of the family Platyrhinidae has been reported only from Bolca and Fayum in Egypt, suggesting similar palaeoecological and palaeoenvironmental features in these two Tethyan areas (Underwood *et al*. 2011; Marramà *et al*. 2018c). This hypothesis is corroborated by the shared presence of small odontaspidid and carcharhinid sharks, which are generalist feeders on small nectobenthic prey and zooplanktivorous coastal bony fishes that represented a relevant trophic resource in the Bolca palaeobiotopes (Marramà *et al*. 2018c).

Conversely, all the specimens of †*Plesiozanobatus egertoni* comb. nov. are from the Pesciara site. The presence of zanobatids in the Pesciara site is consistent with the presence of tropical marine shallow waters hypothesized for the Pesciara palaeobiotope (Marramà *et al.* 2016), because extant panrays inhabit the shallow coastal waters off the eastern central African coast mainly between 10 and 15 m, but also reaching depths of about 100 m (Last *et al*. 2016; Séret 2016).

**Figure 14.**
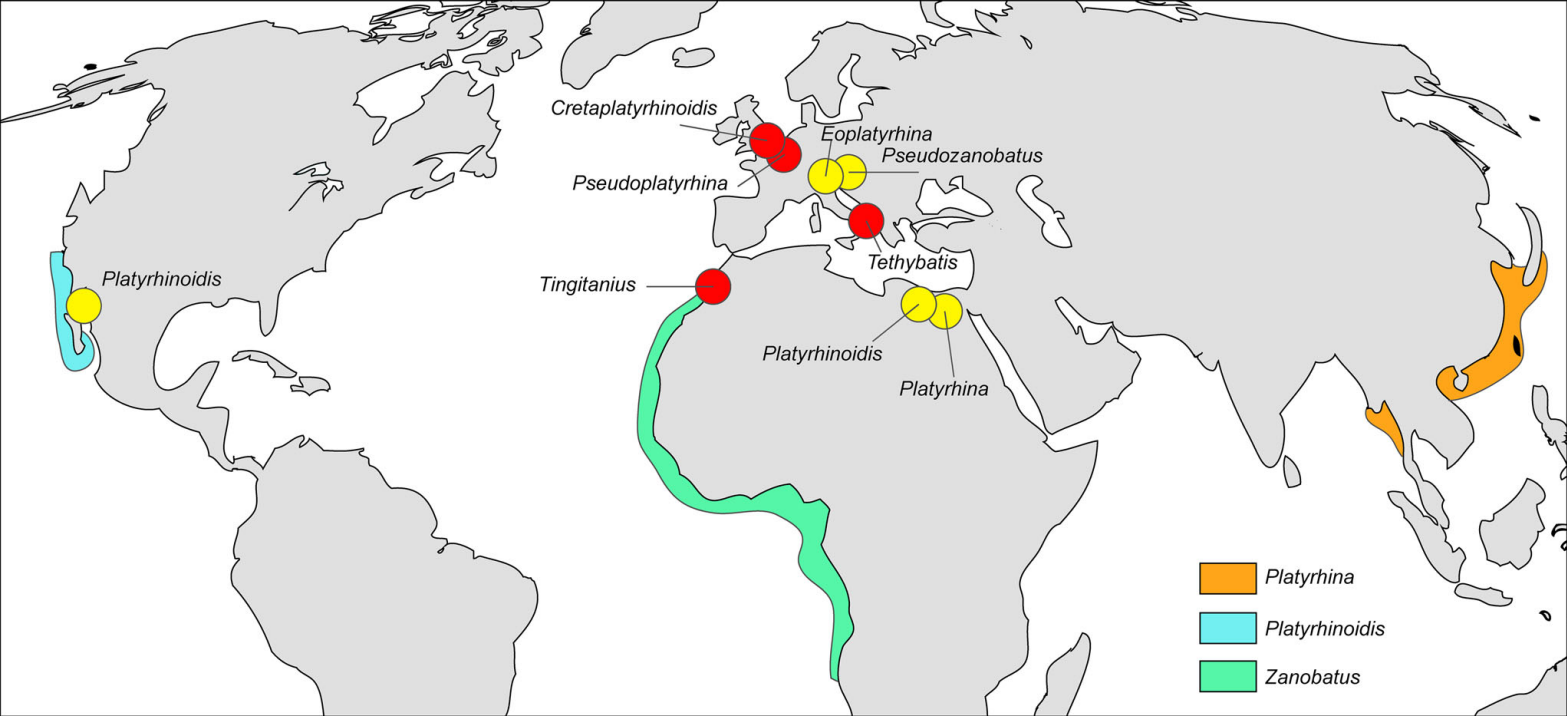
Schematic map showing the Cretaceous (red dots) and Cenozoic (yellow dots) occurrences of platyrhinids and zanobatids. The orange, blue and green colours mark the modern areal distribution of the living species of *Platyrhina*, *Platyrhinoidis* and *Zanobatus*, respectively (data from Last *et al*. 2016).

The fossil record of Platyrhinidae and Zanobatidae is very poor (Fig. 14), but this is likely an artefact, since their teeth might have been misidentified as belonging to *Rhinobatos*, which has been traditionally used as wastebasket genus for many fossil teeth exhibiting a ‘rhinobatoid’ morphology (Kriwet *et al*. 2009; Cappetta 2012; Claeson *et al*. 2013). Fossils of thornback rays and panrays have been reported so far only from the Late Cretaceous to Eocene deposits of the Tethys area, if we exclude a single occurrence from the Pleistocene of California (Fig. 14). Today, platyrhinids are restricted to temperate to tropical marine coastal waters of the north-western and eastern Central Pacific Ocean, whereas zanobatids are only present along the western coast of Africa (Last *et al*. 2016). Molecular analyses suggest that platyrhinids diverged from torpediniforms around 200–175 Ma ago, whereas the clade including *Zanobatus* separated from myliobatiforms around 150 Ma (Aschliman *et al*. 2012b; Bertozzi *et al*. 2016). If these hypotheses are confirmed, it is evident that a large ghost range will characterize the fossil record of these batoid lineages, being the oldest known representatives for platyrhinids and zanobatids of Turonian (*c*. 89 Ma) and Ypresian (*c*. 50 Ma) ages, respectively. The fossil records of both platyrhinids and zanobatids are concentrated in the Tethys, thereby supporting the possibility of a Tethyan origin for these clades, as suggested by Carvalho (2004) and Claeson *et al*. (2013).

## Supplementary Material

Supplemental Material 5

Supplemental Material 4

Supplemental Material 3

Supplemental Material 2

Supplemental Material 1

## Notes

**Associate Editor: Zerina Johanson**
