# Supplementary material for: Revision of the Eocene ‘Platyrhina’ species from the Bolca Lagerstätte (Italy) reveals the first panray (Batomorphii: Zanobatidae) in the fossil record
Source: J Syst Palaeontol. 2020 Jul 13;18(18):1519–42. doi: 10.1080/14772019.2020.1783380 (PMC7455076; doi:10.1080/14772019.2020.1783380)
Supplement: Supplemental Material 1 [file TJSP_A_1783380_SM3990.pdf]

Supplemental material (File 1) to:

Revision of the Eocene '*Platyrhina*' species from the Bolca Lagerstätte (Italy) reveals the first panray (Batomorphii: Zanobatidae) in the fossil record

Giuseppe Marramà[a,b]*, Giorgio Carnevale[a], Kerin M. Claeson[c], Gavin J. P. Naylor[d] and Jürgen Kriwet[b]

[a]*Università degli Studi di Torino, Dipartimento di Scienze della Terra, via Valperga Caluso 35, 10125 Torino, Italy;* [b]*University of Vienna, Department of Palaeontology, Althanstrasse 14, 1090 Vienna, Austria;* [c]*Philadelphia College of Osteopathic Medicine, Philadelphia, PA 19103, USA;* [d]*University of Florida, Florida Museum of Natural History, 1659 Museum Road, 32611 Gainesville, USA*

*Corresponding author. Email: giuseppe.marrama@unito.it

Appendix 1. Character list used for the parsimony analysis. AMC2012 = Aschliman *et al.* (2012); Br2013 = Brito *et al*. (2013); BS1996 = Brito & Seret (1996); Cl2014 = Claeson (2014); CUW 2013 = Claeson *et al*. (2013); Kw2004 = Kriwet (2004); MA2004 = McEachran & Aschliman (2004); MD1998 = McEachran & Dunn (1998); UC2019 = Underwood & Claeson (2019); VS2019 = Villalobos-Segura *et al.* (2019a).

1. Upper eyelid: (0) present, (1) absent (AMC2012, char. 1; VS2019, char. 1).
2. Palatoquadrate: (0) articulates with neurocranium, (1) does not articulate with neurocranium (AMC2012, char. 2; CUW2013, char. 16; VS2019, char. 2).
3. Pseudohyal: (0) absent, (1) present (AMC2012, char. 3; CUW2013, char. 30; VS2019, char. 3)
4. Last ceratobranchial: (0) free, (1) articulates (AMC2012, char. 4; CUW2013, char. 29; VS2019, char. 4).
5. Calcified suprascapulae: (0) absent, (1) present and independent (Based on AMC2012, char. 6; VS2019, char. 6), (2) suprascapular antimeres fused medially (Cl2014, char. 34). **Remarks:** A third state is proposed in comparison to prior versions of the character with takes into account a feature noted in crown Batoidea, where the suprascapular antimeres are fused medially. *Libanopristis* is coded as "?" because in character 23 of Villalobos-Segura *et al*. (2019b), authors were uncertain if the region damaged preserved suprascapulae, despite the similarity to the condition in skates. With the inclusion of outgroups, *Heterodontus* and *Squalus*, new discussion was generated on what homologously was the definition of a suprascapula. Two hypotheses exist. According to Compagno (1999), the scapular process is the unsegmented dorso-medial projection from the scapulocorocoid and articulating with the scapular process might be another small cartilage, the suprascapula. According to Da Silva *et al*. (2018) the projection from the scapulocorocoid as the scapula in sharks (e.g. *Squalus* and *Heterodontus*) with a segmented scapular process, while in batoids, a non-segmented projection is the scapular process. We code two matrices independently for each definition and adjust the correlated characters accordingly.
6. Antorbital cartilage: (0) absent, (1) present (CUW2013, char. 5; VS2019, char. 8).

7. Antorbital cartilage: (0) form a (well-developed, triangular shaped with regular outline), (1) form b (well-developed, variously shaped and with an irregular outline), (2) form c (reduced, triangular shaped and with regular edges) (Modified from CUW2013, char. 6; VS2019, char. 9).
8. Cephalic lobes: (0) absent, (1) present (AMC2012, char. 10; VS2019, char. 10).
9. Cephalic lobes if separated from pectoral fin: (0) form a (single), (1) form b (two lobes) (Modified from AMC2012, char. 10; VS2019, char. 11).
10. Spiracular tentacle: (0) absent, (1) present (AMC2012, char. 12; VS2019, char. 12).
11. Radial cartilages in cauda fin: (0) present, (1) absent (AMC2012, char. 13; VS2019, char. 13).
12. Serrated tail sting: (0) absent, (1) present (AMC2012, char. 14; CUW2013, char. 50; VS2019, char. 14).
13. Placoid scales: (0) absent, (1) present (Modified from AMC2012, char. 15; CUW2013, char. 51; VS2019, char. 15).
14. Alar and malar thorns: (0) absent, (1) present (AMC2012, char. 17; CUW2013, char.48; VS2019, char. 16).
15. Osteodentine: (0) absent, (1) present (Modified from AMC2012, char. 19; CUW2013, char. 19).
16. Osteodentine if present: (0) present in some roots, (1) spread across the teeth (Modified from AMC2012, char. 19; CUW2013, char. 19; VS2019, char. 18).
17. Infraorbital loop of suborbital and infraorbital canals (0) absent, (1) present (AMC2012, char. 21; VS2019, char. 19).
18. Subpleural loop of the hyomandibular canal (0) broad rounded, (1) loop forms a lateral hook, (2) lateral aspects of loop are nearly parallel (AMC2012, char. 22; VS2019, char. 20). **Remark:** This character was rewritten to match the original three states in AMC2012). The coding remains the same.
19. Lateral tubes of subpleural loop: (0) unbranched, (1) branched (AMC2012, char. 23; VS2019, char. 21).
20. Abdominal canal on coracoid bar: (0) absent, (1) present (Modified from (AMC2012, char. 24; VS2019, char. 22).

21. Abdominal canal on coracoid bar: (0) groove – cephalic lateral line forms abdominal canal on coracoid bar, (1) pores (Modified from AMC2012, char. 24; VS2019, char. 23).
22. Scapular loops of scapular canals: (0) absent, (1) present (AMC2012, char. 25; VS2019, char. 24).
23. Hypobranchials: (0) well developed, (1) reduced (VS2019, char. 25).
24. Second hypobranchial-basibranchial: (0) form a (free, they are separated from the basibranchial, (1) form b (the second hypobranchial is fused with the basibranchial), (2) form c (second hypobranchial is articulated with the basibranchial) (VS2019, char. 26).
25. Rostral cartilages: (0) absent, (1) present (Modified form AMC2012, char. 26 and UC2019, char. 1; VS2019, char. 27).
26. Rostral cartilages: (0) reach the tip of the snout, (1) fail to reach the tip of the snout (Modified form AMC2012, char. 26; CUW2013, char. 1; UC2019, char 1 & 2; VS2019, char. 28).
27. Rostral node: (0) expanded laterally, (1) not expanded laterally (AMC2012, char. 27; VS2019, char. 29; modified). **Remarks:** Villalobos-Segura *et al*. (2019a) inverted the original polarity of the states of Aschliman *et al*. (2012) in the character list in their Suppl Material. Moreover, contrary to Aschliman *et al.* (2012), Villalobos-Segura *et al*. (2019a) coded this character also for *Zanobatus* and myliobatiforms even if they lack of rostral cartilage and node. We therefore follow here the original polarity of Aschliman *et al*. (2012) and code inapplicable (-) the character for *Zanobatus* and myliobatiforms.
28. Rostral appendices: (0) absent, (1) present (AMC2012, char. 28; CUW2013, char. 3; VS2019, char. 30).
29. Rostral appendices: (0) form a (calcified), (1) form b (poorly calcified) (VS2019, char. 31).
30. Rostral process: (0) absent, (1) present (AMC2012, char. 29; VS2019, char. 32).
31. Dorsolateral components of the nasal capsule (0) absent, (1) present (AMC2012, char. 30; VS2019, char. 33).
32. Nasal capsules: (0) laterally expanded, (1) ventrolaterally expanded (AMC2012, char. 31; CUW2013, char. 10; VS2019, char. 34).
33. Preorbital process: (0) present, (1) absent (AMC2012, char. 33; VS2019, char.35).

34. Supraorbital crest: (0) present, (1) absent (AMC2012, char. 34; CUW2013, char. 11; VS2019, char. 36).
35. Anterior preorbital foramen: (0) dorsally located, (1) anterior located (AMC2012, char. 35; VS2019, char. 37).
36. Postorbital process: (0) narrow in otic region, (1) reduced/absent, (2) broad and shelf like (AMC2012, char. 36; VS2019, char. 38).
37. Postorbital process: (0) separated from triangular process, (1) fused with triangular process (AMC2012, char. 37; VS2019, char. 39).
38. Postorbital process: (0) projects laterally, (1) projects ventrolaterally (AMC2012, char. 38; VS2019, char. 40).
39. Antimeres of upper and lower jaws: (0) separated, (1) fused (AMC2012, char. 40; VS2019, char. 41).
40. Meckel's cartilage: (0) not expanded laterally, (1) expanded medially (AMC2012, char. 41; VS2019, char. 42).
41. Winglike process on Meckel's cartilage: (0) absent, (1) present (AMC2012, char. 42; VS2019, char. 43).
42. Labial cartilages: (0) present, (1) absent (AMC2012, char. 43; CUW2013, char. 17; VS2019, char. 44). **Remarks:** Contrary to Villalobos-Segura *et al*. (2019a), we coded this character as absent for *Platyrhinoidis* (see Claeson *et al*. 2013). In verifying the coding, we note that *Platyrhina* is absent for labial cartilages in juvenile specimens and could warrant a developmental workup before coding the character as polymorphism. We code *Platyrhina* as present for a labial cartilage.
43. Medial section of hyomandibula: (0) narrow, (1) expanded (AMC2012, char. 44; VS2019, char. 45).
44. Hyomandibula-Meckelian ligament: (0) absent, (1) present (AMC2012, char. 45; VS2019, char. 46).
45. Small cartilages associated with hyomandibular-Meckelian ligament (0) absent, (1) present (AMC2012, char. 47; VS2019, char. 47).

46. Basihyal: (0) absent, (1) present (Modified from AMC2012, char. 48; CUW2013, char. 27; VS2019, char. 48).

47. First hypobranchial: (0) absent, (1) present (Modified from AMC2012, char. 48; CUW2013, char. 27; VS2019, char. 49).

48. Ceratohyal: (0) fully developed, (1) reduced (AMC2012, char. 49; CUW2013, char. 28; VS2019, char. 50).

49. Suprascapula-axial skeleton (0) free of vertebral column, (1) articulates with vertebral column, (2) fused medially to synarcual, (3) fused medially and laterally to synarcual (AMC 2012, char 50; CUW2013, char. 40; VS2019, char. 51). **Remarks:** When using CH coding or DS coding, *Heterodontus* and *Squalus* are coding as (0) or (-) respectively.

50. Orientation of lateral stays: (0) dorsally directed, (1) laterally directed (Modified from AMC2012, char. 51; VS2019, char. 53).

51. Ventral occipital-synarcual articulation: (0) synarcual lip fitted into notch in basicranium, (1) synarcual lip rest in foramen magnum (AMC2012, char. 52; VS2019, char. 54).

52. Second synarcual: (0) absent, (1) present (AMC2012, char. 54; VS2019, char. 55).

53. Scapular process: (0) short, (1) long (AMC2012, char. 56; VS2019, char. 56).

54. Scapular process: (0) without fossa, (1) with fossa (AMC2012, char. 57; VS2019, char. 57).

55. Scapulocoracoid condyles: (0) not horizontal, (1) horizontal (AMC2012, char. 58; VS2019, char. 58).

56. Mesocondyle: (0) equidistant, (1) scapulocoracoid is elongated between the mesocondyle and metacondyle, (2) scapulocoracoid is elongated between the procondyle and the mesocondyle, (3) replaced with a ridge (AMC2012, char. 59; CUW2013, char. 43; VS2019, char. 59).

57. Anterior extension of propterygium: (0) pectoral propterygium fail to reach anterior margin of disc, (1) extend to near the anterior margin of the disc (AMC2012, char. 62; VS2019, char. 60).

58. Distal extent of proximal of propterygium: (0) posterior to mouth, (1) extends between the mouth and antorbital cartilage. (2) reaches antorbital cartilage, (3) reaches nasal capsule (Modified form AMC2012, char. 63; VS2019, char. 61). **Remark:** VS2019 reduced the number of states in this character there were some erroneous codes in AMC2012. We re-divide the derived state and

distinguish the condition relevant to members of Platyrhinidae independent of some stingrays hypothesizing it would potentially be informative for the fossils we describe herein. We do not revert to the original states of AMC2012. We recode stingray taxa, *Urolophus*, *Urotrygon* and *Urobatis* and other taxa based on first hand examination of radiographs, which all have a proximal propterygium that reaches the nasal capsule, surpassing the antorbital cartilage.

59. Proximal section of propterygium: (0) does not surpass the procondyle, (1) extend behind procondyle (AMC2012, char. 64; VS2019, char. 62).

60. Pectoral fin radials: (0) articulate to pterygia, (1) some articulate directly with scapulocoracoid or to the ridge replacing the mesopterygia (AMC2012, char. 65; CUW2013, char. 43; VS2019, char. 63; modified). **Remarks**: The coding for this character has been modified from (0) to (1) for *Zanobatus* since in this taxon, lacking of mesopterygia, some radials articulate directly with the ridge replacing the mesopterygia (pers. obs.; see also McEachran *et al*. 1996).

61. Mesopterygium: (0) present, (1) absent (CUW2013, char. 45; VS2019, char. 64). **Remarks:** we modified the coding of this character from (0) to (1) for *Mobula* since this cartilage is absent/missing also in this taxon (see McEachran *et al*. 1996; Aschliman *et al*. 2012).

62. Pectoral fin radials: (0) not expanded distally, (1) some pectoral fin radials expanded distally (AMC2012, char. 67; VS2019, char. 65).

63. Paired fin rays: (0) aplesodic, (1) plesodic (AMC2012, char. 68; VS2019, char. 66).

64. Puboischiadic bar: (0) plate like, (1) is narrow and moderately to strongly arched without distinct lateral process narrow (2) strongly arched with a triangular medial prepelvic process narrow, (3) moderately arched with a bar like medial prepelvic process (CUW2013, char. 46; VS2019, char. 67).

65. First pelvic radial: (0) band like, (1) slightly expanded distally, articulating with several segments in a parallel fashion, (2) rod-like and articulates with a single radial segment (AMC2012, char. 71; VS2019, char. 68).

66. Pelvic girdle condyles: (0) close together, (1) separated (AMC2012, char. 72; VS2019, char. 69).

67. Clasper length: (0) short, (1) long (AMC2012, char. 73; VS2019, char. 70).

68. Dorsal margin claspers cartilages: (0) lacks medial flange, (1) possesses medial flange (AMC2012, char. 75; VS2019, char. 71).
69. Ventral terminal cartilages: (0) simple, (1) ventral terminal cartilages are free distally and forms components sentinel or is fused with ventral marginal cartilages, (2) ventral terminal cartilages folded ventrally along its long axis to form a convex flange (AMC2012, char. 78; VS2019, char. 73).
70. Ventral terminal cartilages: (0) attached over length to axial cartilages, (1) free of axial (AMC2012, char. 79; VS2019, char. 74).
71. Caudal vertebrae: (0) diplospondylus (1) fused (AMC2012, char. 80; VS2019, char. 75).
72. Ligamentous sling on Meckel's cartilage: (0) absent, (1) present (AMC2012, char. 83; VS2019, char. 76).
73. Depressor mandibularis: (0) present, (1) absent (AMC2012, char. 84; VS2019, char. 77).
74. Spiracularis: (0) undivided, (1) divided (AMC2012, char. 85; VS2019, char. 78).
75. Coracobrachialis: (0) consists of three to five components, (1) single component (AMC2012, char. 87; VS2019, char. 79).
76. Coracohyomandibularis: (0) single origin, (1) separate origins (AMC2012, char. 88; VS2019, char. 80).
77. Arcualia dorsalis: (0) absent, (1) present (Modified from Br2013 char. 30; VS2019, char. 81)
78. Position of vertebral centra in the synarcual relative to position of suprascapula: (0) present through entire length near the articulation with the cranium, (1) reaching rostral to the suprascapula, (2) reaching caudal to the suprascapula (VS2019, char. 82; modified according to Claeson (2011) and personal observation).
79. Nasal capsule margin (0) straight, (1) horn like process (CUW2013, char. 9; VS2019, char. 83; modified). **Remarks**: Contrary to the coding of Claeson *et al.* (2013) we put (?) for *Tethybatis* since, in his original description, Carvalho (2004) pointed out that a horn like process “cannot be seen in the fossil”.
80. Parallel rows of enlarge denticles: (0) absent, (1) present (CUW2013, char. 49; VS2019, char. 84).

81. Ventral antimeres of scapulocoracoid: (0) fused, (1) separate (VS2019, char. 85). **Remarks:** This character is variable during ontogeny for the Torpediniformes, Platyrhinidae, and possibly other batoids. We score this character as polymorphic in the matrix *sensu* VS2019 and also run the analysis without the character in place. It is our opinion that the character is uninformative for Platyrhinidae given the variability during ontogeny and is an example of an area of morphology worthy of developmental study to understand the impact of ontogenetic changes variation and variability on inferring phylogeny.

82. Suprascapula-scapula articulation: (0) curved, (1) crenate/long, (2) crenate/short, (3) ball socket, (4) straight (Modified from AMC2012, char. 53; VS2019, char. 86). **Remarks**: we verified the state in *Zanobatus* as (3) in specimen USNM 193743 and USNM 193991. When using CH coding or DS coding, *Heterodontus* and *Squalus* are coding as (4) or (-) respectively.

83. Differentiated lateral uvulae on teeth: (0) absent, (1) present (CUW2013, char. 22; VS2019, char. 87).

84. Articulation of 2nd hypobranchial with ceratobranchial: (0) present, (1) absent (MD1998, char. 5; VS2019, char. 88). **Remarks:** The original use of this character is from McEachran & Dunn (1998), which states "Anterior portion of second hypobranchial cartilage: (0) present and articulating with second ceratobranchial cartilage; (1) absent and proximal section of second hypobranchial cartilage not articulating with second ceratobranchial cartilage." Within the text, they say Rajidae monophyly is supported by, "(5) anterior portion of second hypobranchial cartilage absent and, thus, second hypobranchial cartilage lacking articulation with second ceratobranchial cartilage (Miyake & McEachran 1991);" Furthermore, the three outgroups MD1998 used were all "guitarfishes" which all have the anterior portion of the 2nd hypobranchial. In our opinion, MD1998, char. 5 should be interpreted in reference to an articulation with the ceratobranchial 2, rather than to the presence or absence of an anterior portion to a 2nd hypbranchial (e.g., VS2019). The secondary character from within their original character about the anterior portion of the second hypobranchial appears to reference a segmented hypobranchial 2, which was not investigated groups other than "guitarfish" and skates by MD1998. The articulation of hypobranchial 2 with ceratobranchial 2 is coded for

Heterodontus and *Pristis* according the Miyake & McEachran (1991). It is parsimony informative as an autoapomorphy of skates.

85. Hypobranchial shape: (0) straight and segmented, (1) loop/horseshoe shaped (BS1996, char. 41), (2) bilateral fused plates, (3) medially fused plates. **Remarks:** According to Brito & Seret (1996) there was no specific tie to the hypobranchial 2 only, it was for all hypobranchials, which can fuse together to be horse-shoe shaped only in "rhinobatoids" (sensu Compagno (1973) in Brito & Seret (1996) - except we don't see it in Compagno (1973))... Miyake & McEachran (1991) does illustrate the morphology for most taxa and the states are derived from those descriptions). We follow the developmental work by Miyake & McEachran (1991), to beginning to describe the segmented nature of the hypobranchials across all batoids. There may be multiple hypobranchial elements that get fused in several Electric rays though they stay bilaterally independent. In stingrays, hypobranchials fuse to a variable degree and form a single plate, most often failing to incorporate the first hypobranchial. We consider this character to be uninformative at this point in time due to lack of developmental information.
86. Rostral dermal denticles: (0) absent, (1) present (Modified from Kw2004, char. 52; VS2019, char. 90).
87. Proximal pectoral elements expanded (Propterygium, Mesopterygium, Metapterygium) distally and paddle-like: (0) absent (1) present (Modified from Kw2004, char 39; VS2019, char. 92).
88. Propterygium-Mesopterygium: (0) form-a differently shaped, (1) form-b similarly shaped (VS2019, char 93).
89. Branchial electric organs: (0) absent, (1) present (AMC2012, char. 86; VS2019, char. 94).
90. Lateral prepelvic process; (0) absent, (1) present (Modified from MD1998 char. 31; VS2019, char. 95).
91. Postpelvic processes: (0) absent, (1) present (BD2004, char. 20; MA2004, char. 63; CUW2013, char. 37). **Remarks**: Contrary to the coding of Claeson *et al.* (2013) we put (?) for *Tethybatis* since in his original description, Carvalho (2004) poited out that “no specific feature of the posterior margin of girdle are preserved”.

92. Ribs: (0) absent; (1) present (new). **Remarks**: based on personal observation and literature we state that ribs are absent in *Chimaera*, *Raja*, *Bathyraja* and all myliobatiforms.

93. Extent of distal pectoral propterygium: (0) not reaching nasal capsules, (1) extending as far as nasal capsules, (2) extending well beyond nasal capsules (BD2004, char. 15; MA2004, char. 55; CUW2013, char. 33)

94. Extent of ectoral radials: (0) not reaching nasal capsules, (1) extending as far as nasal capsules, (2) extending well beyond nasal capsules (BD2004, char. 16; CUW2013, char. 34)

95. Anterior nasal lobe: (0) poorly developed, (1) moderately expanded medially to cover most of the medial half of the naris and extends medially onto the internarial space, (2) extends medially to join its antimere and forms a nasal curtain that falls short of the mouth, (3) extends to or just anterior to the mouth (AMC2012, char. 11)

96. Spiracular tentacle: (0) absent, (1) present (AMC2012, char. 12).

97. Enlarged placoid scales (thorns): (0) absent, (1) present (AMC2012, char. 16)

98. Pulp cavities in tooth roots: (0) large, (1) broad and elongated, (2) small, (3) absent (AMC2012, char. 18)

99. Cephalic lateral line canal on ventral surface: (0) present, (1) absent (AMC2012, char. 20)

100. Jugal arch: (0) absent, (1) present (AMC2012, char. 39)

101. Intermandibularis: (0) present, (1) absent, (2) modified as a narrow band of muscle that originates on the hyomandibula and inserts on the posterior margin of Meckel's cartilage (AMC2012, char. 82).

102. Coracohyoideus: (0) present, (1) absent (AMC2012, char. 89, modified)

103. Coracohyoideus: (0) parallel to body axis, (1) runs parallel to the body axis and is very short, (2) runs diagonally from the wall of the first two gill slits to the posteromedial aspect of the basihyal or first basibranchial, (3) each muscle fuses with its antimere at a raphe near its insertion on the first hypobranchial (AMC2012, char. 89, modified)

104. Radial calcification: (0) crustal, (1) catenated (new). **Remarks**: according to Schaefer & Summers (2005) most of the batoids have pectoral radials highly calcified and completely covered by mineralized tissue (crustal calcification); conversely, modern skates (Rajidae),

urolophids, urotrygonids, dasyatids, and potamotrygonids have low degree of calcification of radials which also have a chain-like appearance (catenated calcification).

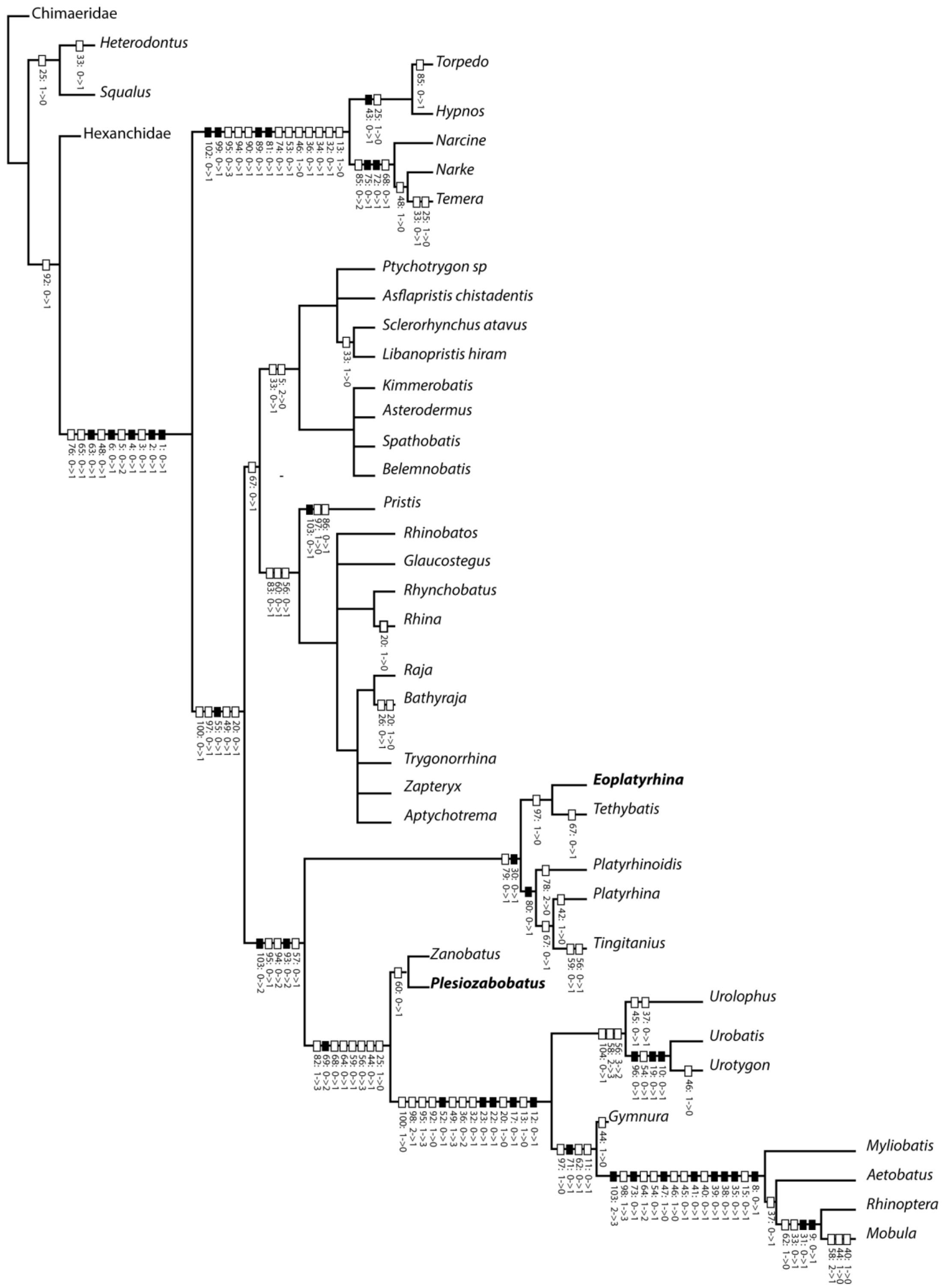

Fig. S1. Character transformations mapped onto strict consensus tree from Fig. 11B. Character transformations having a CI = 1.00 indicated by black boxes and CI < 1.00 indicated by white boxes.

Table S1. Morphometric and meristic data for the three examined specimens of †*Eoplatyrhina bolcensis* (Heckel, 1851) from the Eocene Monte Postale site of the Bolca Lagerstätte.

	MGGC 7449/50			MGP-PD 8873C/8874C			MGP-PD 26279C/26280C		
**Measurements**	**mm**	**% DW**	**% TL**	**mm**	**% DW**	**% TL**	**mm**	**% DW**	**% TL**
Total length	?	?	?	?	?	?	840.3	218.7	100.0
Disc length	354.3	93.4	?	355.7	105.0	?	398.4	103.7	47.4
Disc width	379.4	100.0	?	338.5	100.0	?	384.2	100.0	45.7
Head length (snout to scapulocoracoid)	186.9	49.3	?	186.8	55.1	?	?	?	?
Tail length	?	?	?	?	?	?	441.9	115.0	52.6
Preoral length	70.2	18.5	?	?	?	?	?	?	?
Mouth-scapulocoracoid distance	115.0	30.3	?	?	?	?	?	?	?
Scapulocoracoid width	64.2	16.9	?	62.5	18.5	?	83.4	21.7	9.9
Pelvic girdle width (width across pelvic-fin base)	94.5	24.9	?	?	?	?	100.6	26.2	12.0
Pelvics to tip of tail length	?	?	?	?	?	?	393.7	102.5	46.9
Neurocranial length	130.9	34.5	?	128.6	38.00	?	?	?	?
Distance from tip of disc to max width disc	208.0	54.8	?	197.5	58.4	?	224.0	58.3	26.7
Eye diameter	?	?	?	12.1	3.6	?	?	?	?
Interorbital width	?	?	?	40.8	12.1	?	?	?	?
Snout (preorbital) length	?	?	?	73.1	21.6	?	?	?	?
Pelvic-fin length (excl. clasper)	76.6	20.2	?	80.2	23.7	?	92.3	24.0	11.0
Snout to pelvic-fin origin	315.2	83.1	?	306.8	90.6	?	371.2	96.6	44.2
Clasper length	74.5	19.6	?	76.8	22.7	?	83.1	21.6	9.9
First dorsal-fin base length	?	?	?	30.7	9.1	?	39.4	10.3	4.7
Second dorsal-fin base length	?	?	?	32.4	9.6	?	42.7	11.1	5.1
Snout to first-dorsal fin origin	?	?	?	531.9	157.1	?	600.0	156.2	71.4
Snout to second-dorsal fin origin	?	?	?	599.4	177.1	?	660.3	171.9	78.6
Interdorsal distance	?	?	?	40.2	11.9	?	?	?	?

Caudal-fin length	?	?	?	?	?	?	91.3	23.8	10.9
**Meristics**									
Propterygial radials		38			35			36	
Mesopterygial radials		8-9			8-10			10	
Metapterygial radials		38-40			38			41	
Total pectoral radials		84-86			81-83			87	
Pelvic radials		18-20			18-20			21	
Total caudal-fin radials (ventral+dorsal)		?			?			40-50	
Trunk vertebrae		24			20			22	
Vertebrae from pelvic girdle to caudal-fin origin		90			95			90	
Caudal vertebrae		?			?			23	
Total vertebrae		?			?			132	
Rib pairs		15			15			16	

Table S2. Morphometric and meristic data for the six examined specimens of †*Plesiozanobatos egertoni* (De Zigno, 1876) from the Eocene Pesciara site of the Bolca Lagerstätte.

	MGP-PD 154Z			MB.f 1608.1/2			MCSNV IG.43347		
**Measurements**	**mm**	**% DW**	**% TL**	**mm**	**% DW**	**% TL**	**mm**	**% DW**	**% TL**
Total length	481.2	157.1	100.0	426.2	146.2	100.0	479.8	170.5	100.0
Disc length	340.2	111.0	70.7	286.0	98.1	67.1	307.6	109.3	64.1
Disc width	306.4	100.0	63.7	291.6	100.0	68.4	281.4	100.0	58.6
Head length (snout to scapulocoracoid)	106.8	34.9	22.2	146.3	50.2	34.3	153.3	54.5	32.0
Tail length	?	?	?	189.0	64.8	44.3	222.5	79.1	46.4
Scapulocoracoid width	135.5	44.2	28.2	?	?	?	?	?	?
Pelvic girdle width (width across pelvic-fin base)	?	?	?	62.9	21.6	14.8	64.8	23.0	13.5
Distance from tip of disc to max width disc	?	?	?	142.4	48.8	33.4	159	56.5	33.1
Pelvic-fin length (excl. clasper)	?	?	?	?	?	?	74.0	26.3	15.4
First dorsal-fin base length	?	?	?	31.4	10.8	7.4	?	?	?
Second dorsal-fin base length	?	?	?	31.0	10.6	7.3	?	?	?
Snout to first-dorsal fin origin	?	?	?	330.9	113.5	77.6	?	?	?
Snout to second-dorsal fin origin	?	?	?	367.0	125.9	86.1	?	?	?
Interdorsal distance	?	?	?	9.0	3.1	2.1	?	?	?
Caudal-fin length	?	?	?	?	?	?	44.3	15.7	9.2
**Meristics**									
Total pectoral radials		70			75			?	
Pelvic radials		?			?			?	
Total vertebrae		?			80			80-90	
Rib pairs		?			?			?	

Table S2. (continues…)

	MCSNV IG.142530			MCSNV VII.B.80/81			MCSNV VII.B.88/89		
**Measurements**	**mm**	**% DW**	**% TL**	**mm**	**% DW**	**% TL**	**mm**	**% DW**	**% TL**
Total length	524.5	156.0	100.0	1149.3	153.4	100.0	506.3	162.4	100.0
Disc length	?	?	?	639.1	85.3	55.6	308.5	99.0	60.9
Disc width	336.3	100.0	64.1	749.2	100.0	65.2	311.7	100.0	61.6
Head length (snout to scapulocoracoid)	?	?	?	368.2	49.2	32.0	143.4	46.0	28.3
Tail length	203.0	60.4	38.7	569.3	76.0	49.5	235.3	75.5	46.5
Scapulocoracoid width	?	?	?	?	?	?	120.2	38.6	23.7
Pelvic girdle width (width across pelvic-fin base)	?	?	?	195.8	26.1	17.0	?	?	?
Distance from tip of disc to max width disc	?	?	?	?	?	?	?	?	?
Pelvic-fin length (excl. clasper)	?	?	?	171.0	22.8	14.9	100.4	32.2	19.8
First dorsal-fin base length	?	?	?	85.9	11.5	7.5	?	?	?
Second dorsal-fin base length	?	?	?	74.2	9.9	6.5	?	?	?
Snout to first-dorsal fin origin	?	?	?	874.7	116.8	76.1	?	?	?
Snout to second-dorsal fin origin	?	?	?	977.5	130.5	85.1	?	?	?
Interdorsal distance	?	?	?	18.3	2.4	1.6	?	?	?
Caudal fin length	?	?	?	?	?	?	?	?	?
**Meristics**									
Total pectoral radials		?			70-75			65	
Pelvic radials		?			20			20	
Total vertebrae		?			?			?	
Rib pairs		?			10			?	

Table S3. Morphological and meristic characters useful to distinguish †*Eoplatyrhina bolcensis* (Heckel, 1851) and †*Plesiozanobatos egertoni* (De Zigno, 1876) from other living and fossil representatives of the Platyrhinidae and Zanobatidae, respectively. All body proportions as percentage of total length (%TL). Data from Carvalho (2004), Iwatsuki *et al*. (2011), Claeson *et al*. (2013), Last *et al*. (2016), White & Last (2016), Séret (2016), Hall *et al*. (2018) and from the extant comparative material examined. En dash (-) indicates that data are not relevant or inapplicable for the taxon.

	PLATYRHINIDAE					ZANOBATIDAE	
	**†*Eoplatyrhina***	***Platyrhina***	***Platyrhinoidis***	**†*Tingitanius***	**†*Tethybatis***	**†*Plesiozanobatus***	***Zanobatus***
**Morphological features**							
Propterygial radials	35-38	22-34	23-26	?	?	?	?
Mesopterygial radials	8-10	7-12	6-7	?	?	-	-
Metapterygial radials	38-41	19-23	17-20	?	?	?	?
Total pectoral radials	81-87	55-66	46-52	?	45	65-75	77-86
Pelvic radials	18-21	18-19	16-18	?	15	20	?
Trunk vertebrae (excluding synarcual)	20-24	23-25	17-18	?	41-44	?	21-31
Vertebrae from pelvic girdle to caudal-fin origin	88-95	69-81	78-82	?	57-61	?	?
Caudal vertebrae	23	35-44	25-30	?	?	?	?
Total vertebrae	about 132	125-139	120-130	?	101-102	80-90	129-153
Rib pairs	15-16	10-12	13-14	?	8	10	?
Rostral cartilage	long	short	long	?	long	-	-
Anterior fontanel shape	isosceles triangle	oval	eight figure	isosceles triangle	?	-	-
Nasal capsule horn	present	present	present	present	absent ?	-	-
Space between hyomandibulae and jaws	large	small	small	small ?	large	-	-
Labial cartilages	?	present	absent	absent	?	-	-
Clasper length	short	long	short	long	long	-	-
Thorns	absent	present	present	present	absent	-	-

Body proportions (as %TL)							
Disc length	47	40-52	36-38	?	?	56-71	48-56
Disc width	46	44-58	45-47	?	?	58-68	53-65
Head length	?	17-29	19-21	?	?	22-34	24-31
Snout to first dorsal length	71	59-71	58-61	?	?	76-78	63-75
Snout to second dorsal length	79	70-78	67-70	?	?	85-86	74-86
Snout to pelvic origin length	44	36-40	31-32	?	?	?	41-49
Tail length	53	52-64	64-68	?	?	39-50	49-54
Clasper length	10	7-22	?	?	?	?	?

Supplementary references

Aschliman, N. C., Claeson, K. M. & McEachran, J. D. 2012. Phylogeny of Batoidea. Pp. 57–96 in J. C. Carrier, J. A. Musick & M. R. Heithaus (eds) *Biology of sharks and their* relatives. 2nd ed. CRC Press, Boca Raton.

Brito, P. M. & Seret, B. 1996. The new genus *Iansan* (Chondrichthyes, Rhinobatoidea) from the Early Cretaceous of Brazil and its phylogenetic relationships. Pp. 47–52 in G. Arratia & G. Viohl (eds) *Mesozoic fishes: systematics and paleoecology: proceedings of the international meeting Eichstätt*. Verlag Dr. Friedrich Pfeil, Munich.

Brito, P. M., Leal, M. E. C. & Gallo, V. 2013. A new Lower Cretaceous guitarfish (Chondrichthyes, Batoidea) from the Santana Formation, Northeastern Brazil. *Boletim do Museu Nacional, Geologia*, **75**, 1–13.

Carvalho, M. R. de. 2004. A Late Cretaceous thornback ray from southern Italy, with a phylogenetic reappraisal of the Platyrhinidae (Chondrichthyes: Batoidea). Pp. 75–100 in G. Arratia & A. Tintori (eds) *Mesozoic fishes 3 – Systematics, Paleoenvironments and Biodiversity.* F. Pfeil-Verlag, München.

Claeson, K. M. 2011. The synarcual cartilage of batoids with emphasis on the synarcual of Rajidae. *Journal of Morphology*, **272**, 1444–1463.

Claeson, K. M. 2014. The impacts of comparative anatomy of electric rays (Batoidea: Torpediniformes) on their systematic hypotheses. *Journal of Morphology*, **275**, 597–612.

Claeson, K. M., Underwood, C. J. & Ward, D. J. 2013. *Tingitanius tenuimandibulus*, a new platyrhinid batoid from the Turonian (Cretaceous) of Morocco and the Cretaceous radiation of the Platyrhinidae. *Journal of Vertebrate Paleontology*, **33**, 1019–1036.

Compagno, L. J. V. 1999. Chapter 3. Endoskeleton. Pp. 69–92 in W.C. Hamlett (ed.) Sharks, skates and rays. *The biology of elasmobranch fishes*. Johns Hopkins Press, Maryland.

Da Silva, J. P. C. B, Vaz, D. F. B. & Carvalho M. R. 2018. Phylogenetic inferences on the systematics of squaliform sharks based on elasmobranch scapular morphology (Chondrichthyes: Elasmobranchii*). Zoological Journal of the Linnean Society*, **182**, 614–630.

Garman, S. 1913. *The Plagiostomia (Sharks, Skates and Rays). Memoirs of the Museum of Comparative Zoology at Harvard College, 36*. 528 pp.

Hall, K. C., Hundt, P. J., Swenson, J. D., Summers, A. P. & Crow, K. D. 2018. The evolution of underwater flight: The redistribution of pectoral fin rays, in manta rays and their relatives (Myliobatidae). *Journal of Morphology*, **279**, 1155–1170.

Iwatsuki, Y., Miyamoto, K., Nakaya, K. & Zhang, J. 2011. A review of the genus *Platyrhina* (Chondrichyhys: Platyrhinidae) from the northwestern Pacific, with descriptions of two new species. *Zootaxa*, **2738**, 26–40.

Kriwet, J. 2004. The systematic position of the Cretaceous sclerorhynchid sawfishes (Elasmobranchii, Pristiorajea). Pp. 57–73 in G. Arratia & A. Tintori (eds) *Mesozoic fishes 3 – systematics, paleoenviroments and biodiversity: proceedings of the international meeting Serpiano.* Verlag Dr. Friedrich Pfeil, Munich.

Last, P. R., White, W., Carvalho, M. R., Séret, B., Stehmann, M. & Naylor, G. J. P. 2016. *Rays of the world*. CSIRO Publishing, Clayton North, 790 pp.

McEachran, J. D. & Aschliman, N. 2004. Phylogeny of Batoidea. Pp. 79–113 in J. C. Carrier, J. A. Musick & M. R. Heithaus (eds) *Biology of sharks and their relatives*. CRC Press, Boca Raton.

McEachran, J. D. & Dunn, K. A. 1998. Phylogenetic analysis of skates, a morphologically conservative clade of elasmobranchs (Chondrichthyes: Rajidae). *Copeia*, **1998**, 271–290.

McEachran, J. D., Dunn, K. A. & Miyake, T. 1996. Interrelationships of the batoid fishes (Chondrichthyes: Batoidea). Pp. 63–84 in M. L. J. Stassney, L. R. Parenti & G. D. Johnson (eds) *Interrelationships of fishes*. Academic Press, San Diego.

Miyake, T. & McEachran, J. D. 1991. The morphology and evolution of the ventral gill arch skeleton in batoid fishes (Chondrichthyes: Batoidea). *Zoological Journal of the Linnean Society*, **102**, 75–100.

Schaefer, J. T. & Summers, A. P. 2005. Batoid wing skeletal structure: novel morphologies, mechanical implications, and phylogenetic patterns. *Journal of Morphology*, **264**, 298–313.

Séret, B. 2016. *Zanobatus maculatus*, a new species of panray from the Gulf of Guinea, eastern central Atlantic (Elasmobranchii: Batoidea: Zanobatidae). *Zootaxa*, **4161**, 509–522.

Underwood, C. J. & Claeson, K. M. 2019. The Late Jurassic ray *Kimmerobatis etchesi* gen. et sp. nov. and the Jurassic radiation of the Batoidea. *Proceedings of the Geologists' Association*, **130**, 345–354.

Villalobos-Segura, E., Underwood, C. J., Ward, D. J. & Claeson, K. M. 2019a. The first three-dimensional fossils of Cretaceous sclerorhynchid sawfish: *Asflapristis cristadentis* gen. et sp. nov., and implications for the phylogenetic relations of the Sclerorhynchoidei (Chondrichthyes). *Journal of Systematic Palaeontology*, 17, 1847–1870.

Villalobos-Segura, E., Underwood, C. J. & Ward, D. J. 2019b. The first skeletal record of the enigmatic cretaceous sawfish genus *Ptychotrygon* (Chondrichthyes, Batoidea) from the Turonian of Morocco. *Papers in Palaeontology*. doi: 10.1002/spp2.1287

White, W. T. & Last, P. R. 2016. *Platyrhina psomadakisi* sp. nov., a new species of fanray (Batoidea: Platyrhinidae) from the Andaman Sea, the first record of this family in the Indian Ocean. *Zootaxa*, **4121**, 533–544.
